# Hydrogel-extracellular vesicle engineering delivery system: a promising therapeutic strategy for wound healing

**DOI:** 10.1186/s12951-026-04236-1

**Published:** 2026-03-11

**Authors:** Fengrui Gao, Zhuang Hu, Huazhen Xu, Yao Yu, Shan Gao, Jiawen Sun, Guonong He, Xin Peng

**Affiliations:** 1 Ningbo Municipal Hospital of Traditional Chinese Medicine (TCM), Affiliated Hospital of Zhejiang Chinese Medical University, Ningbo, 315016 China; 2 Department of Neurosurgery, Johns Hopkins University School of Medicine, Baltimore, MD 21205 USA; 3 College of Pharmaceutical Sciences, Zhejiang Chinese Medical University, Hangzhou, 311400 China

## Abstract

Chronic refractory wounds, such as diabetic foot ulcers, present significant clinical challenges due to a dysregulated healing microenvironment. Extracellular vesicles (EVs) have emerged as promising therapeutic agents owing to their pro-angiogenic, anti-inflammatory, and regenerative properties. However, their clinical translation is hampered by rapid clearance and instability at the wound site. Hydrogel-based delivery systems offer an effective strategy to overcome these limitations by providing a protective and tunable platform for sustained EVs release. This review systematically synthesizes contemporary advances in EVs hydrogel (EVH) systems for chronic wound therapy. We critically evaluate design strategies encompassing various hydrogel matrices (natural, synthetic, and smart responsive), engineering approaches for EVs modification, and controlled-release mechanisms that collectively enhance therapeutic efficacy. By integrating findings from preclinical studies across diverse wound models, we highlight the synergistic roles of EVH systems in promoting angiogenesis, modulating immune responses, and accelerating tissue regeneration. Furthermore, this review addresses key translational challenges, including scalable EVs production, standardization, biosafety, and regulatory pathways. Finally, we provide forward-looking perspectives on the clinical translation of next-generation, intelligent EVH systems, aiming to bridge the gap between innovative design and practical therapeutic application.

**Graphical Abstract:**

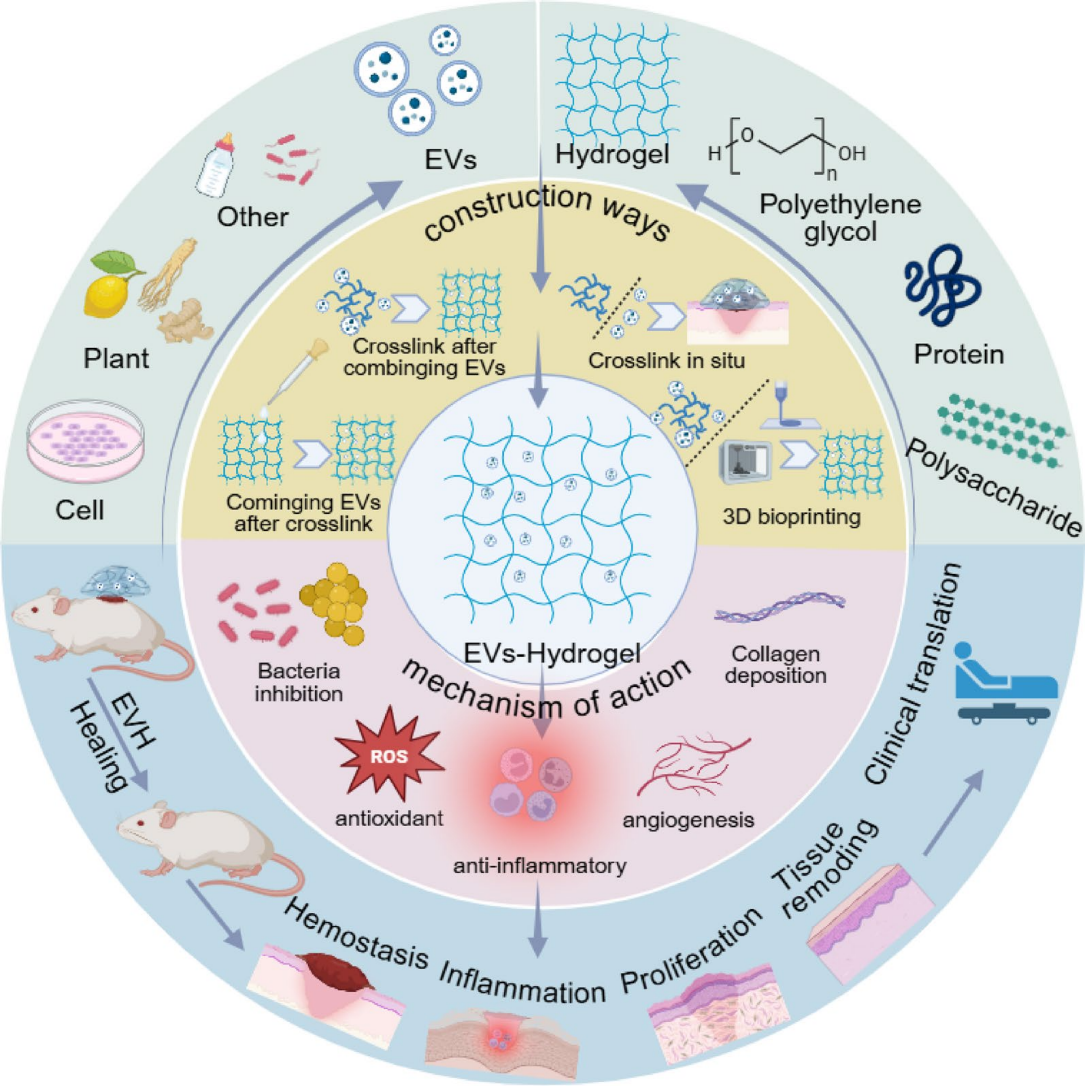

## Introduction

Chronic non-healing wounds, such as diabetic foot ulcers and radiation injuries, pose significant clinical challenges due to dysregulation within their healing microenvironment. These conditions frequently result in diminished quality of life for patients, substantial healthcare costs, and severe consequences including amputation. Traditional wound management strategies, including debridement, dressing changes, and growth factor therapies, while demonstrating some efficacy, often prove limited in regulating complex pathophysiological processes [1, 2]. In recent years, extracellular vesicles (EVs) have emerged as a novel acellular therapeutic strategy, demonstrating substantial potential in tissue repair due to their inherent pro-angiogenic, anti-inflammatory, and regenerative properties [3, 4]. EVs are nanoscale bilayer lipid vesicles actively secreted by cells, carrying bioactive substances such as proteins, nucleic acids, and lipids. They function as crucial intercellular messengers, coordinating multiple healing stages including inflammation resolution, matrix remodelling, and tissue regeneration [5, 6]. Given the heterogeneity of EVs in terms of size, biogenesis and molecular contents, coupled with the difficulty of current isolation techniques in enriching specific subpopulations, their study and application present challenges. In accordance with the MISEV 2023 guidelines, this review will uniformly employ the generic term EVs [7].

However, the clinical application of free EVs faces a series of bottlenecks: they are rapidly cleared in vivo, susceptible to enzymatic degradation, exhibit short retention times at target sites, and lack the capacity for active response to the dynamic wound microenvironment [8, 9]. To overcome these limitations, biomaterial delivery systems, particularly hydrogels, offer a highly promising solution. Hydrogels constitute three-dimensional network structures formed by cross-linking hydrophilic polymers, capable of absorbing and retaining substantial water content while mimicking the physical properties of the natural extracellular matrix (ECM) [10]. Serving as carriers for EVs, hydrogels not only provide physical protection against EVs degradation but also significantly prolong the duration of EVs action at the wound site through controlled release mechanisms, enabling localised high-concentration delivery [11, 12].

With the convergence of materials science and regenerative medicine, hydrogel carrier design has evolved from simple passive “reservoirs” to sophisticated “active therapeutic platforms”. On the one hand, hydrogel systems based on natural polymers (such as hyaluronic acid, chitosan, collagen, alginate) and synthetic polymers (such as polyethylene glycol, polyvinyl alcohol) have been extensively studied due to their tunable biocompatibility, degradability, and mechanical properties [13–15]. Concurrently, smart responsive hydrogels have emerged as a promising development. These materials can detect specific pathological signals within the wound microenvironment, including changes in pH, reactive oxygen species (ROS), matrix metalloproteinase levels, or glucose concentration. By undergoing controlled physicochemical transformations in response, they achieve spatiotemporal and on-demand release of EVs, thereby substantially improving therapeutic precision [16–18]. Furthermore, integrating functional components such as antimicrobial, antioxidant, pro-angiogenic, or conductive agents into hydrogel networks enables the construction of multifunctional synergistic systems. These systems not only deliver EVs but also actively intervene in the wound microenvironment, producing synergistic therapeutic effects where the whole is greater than the sum of its parts. Examples include concurrently clearing infections, alleviating oxidative stress, and promoting tissue regeneration[19–22].

EVs derived from a variety of cellular sources, including mesenchymal stem cells, endothelial cells, and M2 macrophages, as well as from emerging non-mammalian sources such as plant exosomes, exhibit distinct biological signatures and functional specializations. This diversity offers a rich array of therapeutic options for wounds exhibiting varied pathological characteristics [3, 23]. Integrating functionalised EVs from specific sources with bespoke hydrogel platforms represents a cutting-edge approach in developing personalised wound treatment strategies [1, 24].

Despite encouraging results from preclinical studies, the EVs hydrogel (EVH) systems faces multiple challenges in advancing towards clinical translation [25]. These include the large-scale standardised production and quality control of EVs, the long-term stability and sterilisation processes of EVH composites, insufficient translational relevance of preclinical models, and complex regulatory pathways and safety assessments [9, 26, 27].

This review aims to systematically collate and critically analyse recent advances in EVH systems for chronic wound treatment. We shall first outline the biological characteristics of EVs and the design principles for hydrogels as delivery platforms. Subsequently, we will explore the construction, release behavior, therapeutic mechanisms, and optimization strategies of EVH systems. This discussion will focus on EVs derived from diverse sources, placing particular emphasis on those from adipose-derived stem cells (ADSCs), human umbilical cord mesenchymal stem cells (HUMSCs), macrophages and bone marrow-derived mesenchymal stem cells, and plants. Finally, from a translational medicine perspective, this paper will focus on analysing the core challenges currently facing the field, encompassing production standardisation, safety assessment, clinical translation pathways, and commercialisation prospects, while offering forward-looking insights for future development. This aims to provide a clear roadmap for advancing the next generation of efficient, intelligent EVH systems from the laboratory to clinical application.

## Overview of EVs and EVH system construction

**Fig. 1:**
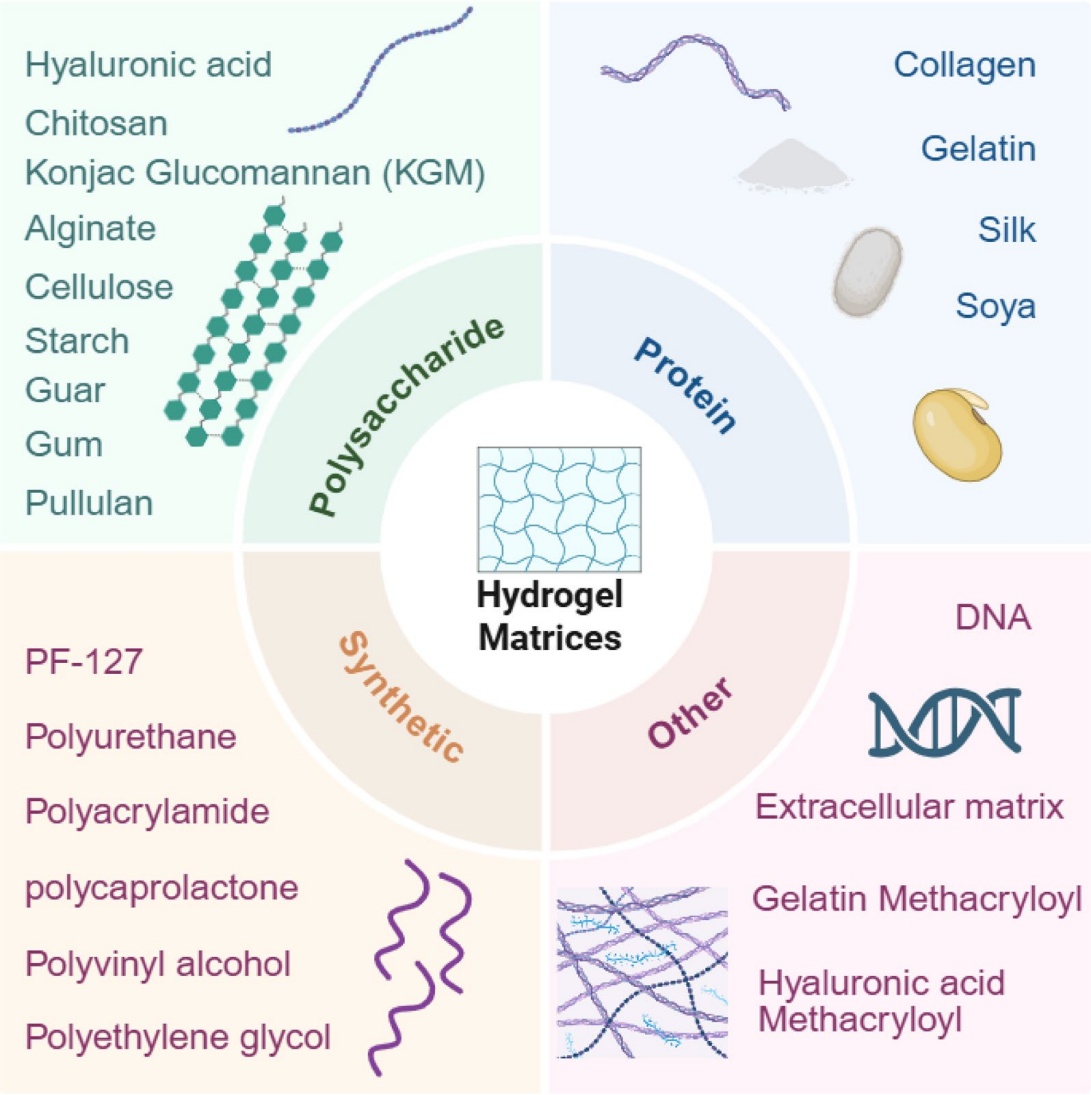
Schematic diagram of hydrogels origin(Figure created using BioRender)

Hydrogels are three-dimensional network structures formed by cross-linking hydrophilic polymers, capable of absorbing and retaining substantial amounts of water without dissolving themselves. This property renders them an ideal carrier for delivering EVs: their highly hydrated matrix protects EVs from degradation, while the controlled release network significantly prolongs the retention and action time of EVs at the wound site, thereby overcoming the bottleneck of free EVs being rapidly cleared [28]. Based on origin, hydrogels are primarily categorised into natural and synthetic types (Fig. 1). Natural hydrogels (e.g., polysaccharides, proteins) possess inherent biological activity and excellent biocompatibility. synthetic hydrogels (e.g., PEG, PVA) offer advantages in precisely engineered structures and uniform, stable properties. To maximise the therapeutic potential of EVs, hydrogel carriers must be selected based on the specific pathological characteristics of the wound microenvironment (e.g., infection, high oxidative stress, impaired glucose metabolism).

### Rational selection and design of hydrogels

#### Selection principles

Selecting an appropriate hydrogel carrier for EVs hinges on precisely matching the material’s physicochemical and biological properties to the pathophysiological requirements of the target wound. Hydrogels from different sources exhibit significant variations in biocompatibility, degradation behaviour, mechanical strength, and inherent biological activity (Table 1). These characteristics directly determine the loading efficiency, activity retention, release kinetics, and ultimate therapeutic efficacy of EVs within the dynamic wound environment [29–31].

**Table 1:** Comparative characteristics and selection guidelines for different types of hydrogels used in controlled-release systems for EVs

Characteristic Dimension	Natural Polysaccharides	Natural Proteins	Synthetic	Hybridisation
Examples of typical materials	Hyaluronic acid [32, 33] Chitosan [34, 35, 36, 37] Alginate [38, 39, 40]	Collagen [41, 42] Gelatin [43, 44] Sericin [45, 46]	Polyethylene glycol [47] Polyvinyl alcohol [48, 49] Polyacrylamide [50]	Methacrylated gelatin [51] Chitosan-PEG composite [52]
Biocompatibility	Excellent	Excellent	Good	Excellent
Degradability	Rapid, enzyme- or ion-controlled degradation	Enzymatically degradable, with adjustable degradation rates	Can be engineered for non-degradation or controlled hydrolytic degradation	Designable, incorporating dual-component properties
Mechanical properties	Typically weak, requiring cross-linking for reinforcement	Collagen/gelatin offer greater softness. sericin provides high strength and superior toughness	Highly adjustable with broad range	Superior comprehensive performance with broad tunability range
Intrinsic biological activity	Anti-inflammatory, pro-angiogenic, antimicrobial, immunomodulatory	Rich in cell adhesion sites, directly supporting cell migration and proliferation	Typically biologically inert	This depends on its natural ingredients

In practical applications, selection requires systematic consideration of the wound’s specific pathological state (such as the presence of infection, ischaemia, hyperglycaemia, or excessive inflammation) and the anticipated therapeutic objectives (rapid vascularisation, immunomodulation, or anti-scarring). This enables the screening or design of the most suitable EVs hydrogel carrier from the categories.

#### Smart responsive hydrogels

Conventional hydrogels rely primarily on passive diffusion or matrix degradation for EVs release, lacking the capacity for active response and regulation within the complex dynamic microenvironment of wounds. Smart responsive hydrogels can detect specific local wound pathophysiological signals (such as pH, ROS, enzyme activity, glucose concentration, etc.) and achieve spatiotemporal controlled release of encapsulated EVs through controllable transformations of their own physicochemical structure (Table 2). Such systems not only enhance EVs utilisation but also dynamically adapt to different stages of wound healing, making them particularly suitable for chronic wounds with complex and variable microenvironments, such as diabetic wounds [53, 54].

**Table 2:** Comparative characteristics of four categories of smart responsive hydrogels for diabetic wound healing

Response signals	Core Materials	Release Mechanism	Ref.
**pH**	•Polymers containing ionisable groups •dynamic covalent bonds (e.g., Schiff base)	•Protonation of functional groups in acidic environments, gel swelling or network disruption, accelerates release. release slows when pH returns to neutral	[55–60]
**ROS**	•Containing ROS-sensitive bonds (phenylboronic ester bond, thio-ketone bond) •often combined with natural antioxidants (e.g., tannic acid)	•Achieve precise delivery to regions experiencing oxidative stress. The release process also facilitates synergistic antioxidant therapy	[17, 61, 62]
**Enzyme**	•Enzyme-specific substrate peptide sequences embedded within crosslinked networks	•Enzymatic cleavage of substrate peptides enables gel-specific degradation, achieving localised, time-controlled drug release	[63, 64]
**Glucose**	•Phenylboronic acid (PBA) and its derivatives •glucose oxidase (GOX) system	•PBA type: Glucose binding induces gel swelling/charge alteration, accelerating release •GOX type: Catalyses acid production, inducing pH decrease to trigger secondary response release	[65–68]

Based on the properties, smart responsive hydrogels can achieve on-demand release of EVs at diabetic wound sites through targeted signal-responsive mechanisms, thereby providing a more precise therapeutic strategy for chronic, difficult-to-heal wounds.

#### Synergistic strategies for multifunctional hydrogels

In chronic wound therapy, an ideal hydrogel carrier should transcend the role of a passive “reservoir” for EVs. Through sophisticated material design, integrating inherent therapeutic activity with enhanced clinical applicability to construct multifunctional synergistic systems is pivotal to elevating the overall efficacy and clinical translation potential of EVs-based therapies. This strategy aims to transform the hydrogel itself into an active therapeutic participant, generating a synergistic effect with EVs where the whole is greater than the sum of its parts [69, 70].

In summary, the synergistic strategy of multifunctional hydrogels centres on the profound integration of ‘therapeutic functions’ with ‘delivery functions’. Hydrogels thus evolve from simple encapsulation materials into ‘active therapeutic platforms’. Not only does it provide protection and controlled release for EVs, but it also actively modulates the wound microenvironment (e.g., anti-infection, anti-oxidation) and ensures efficient, precise delivery of therapeutic units through intelligent engineered properties (e.g., injectability, self-repair, strong adhesion).

 By significantly enhancing the EVH system's capacity to address complex chronic wound pathologies, this integrated design offers a pivotal strategy to accelerate its clinical translation

### Overview of EVs

EVs are actively secreted, lipid bilayer-enclosed nanoscale particles that serve as pivotal messengers in intercellular communication. They carry biologically active cargo such as proteins, nucleic acids, and lipids, transferring these to recipient cells to regulate target cell physiology and pathological processes. In wound healing, EVs demonstrate significant potential in coordinating the resolution of inflammation, matrix remodelling, and tissue regeneration by transmitting pro-angiogenic, anti-inflammatory, and pro-regenerative signals.

#### Biological characteristics and isolation strategies of EVs

**Fig. 2:**
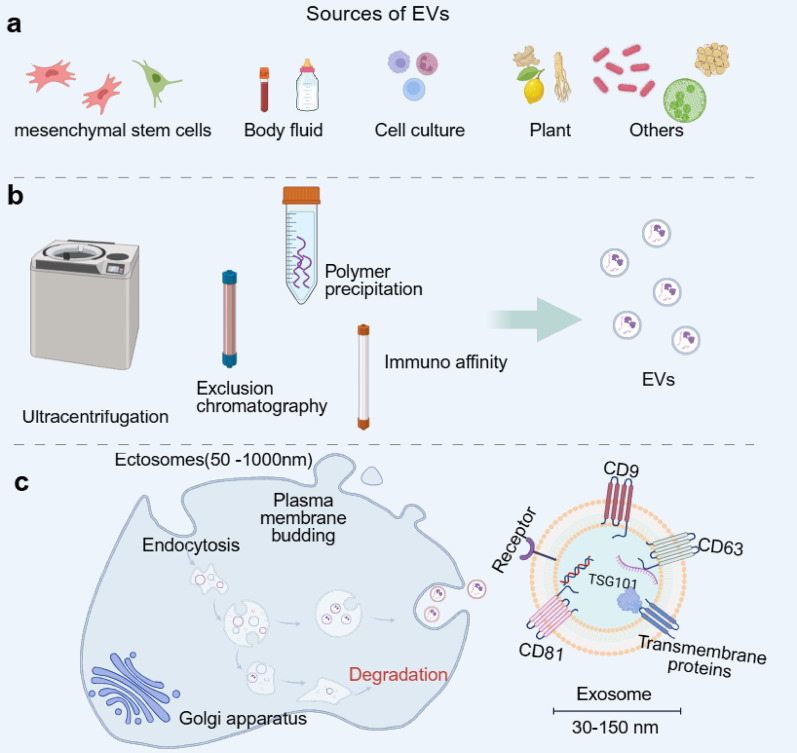
Schematic diagram of the (a)sources, (b)extraction and (c)bioproduction processes of EVs(Figure created using BioRender) https://BioRender.com

Based on their biogenesis pathways and physical dimensions, EVs can be primarily categorised into distinct subpopulations with varying origins and functions, including exosomes, microvesicles, and apoptotic bodies. Among these, the formation of exosomes (diameter 30–150 nm) is most representative: originating from endosomes formed by invagination of the plasma membrane, they mature through a multivesicular stage before ultimately being released extracellularly via fusion with the plasma membrane (Fig. 2c). This finely regulated process determines the specific proteins, nucleic acids, and lipids constituting the ‘cargo’ carried by EVs. This enables them to transmit complex biological information to recipient cells, thereby regulating diverse physiological and pathological processes such as tissue repair and immune responses [71].

The sources of EVs are exceptionally diverse, providing a rich foundation of options and functional capabilities for therapeutic applications. They are secreted by virtually all types of mammalian cells, such as mesenchymal stem cells with multi-lineage differentiation potential, functionally plastic macrophages, and endothelium cells involved in vascular construction, and are also found naturally in various bodily fluids including blood and urine [72, 73] (Fig. 2a). In recent years, non-mammalian-derived vesicles such as plant-derived EVs and bacterial membrane vesicles have garnered attention. Their widespread availability, relatively low preparation costs, and potentially distinct immunogenic characteristics have expanded the resource dimensions for therapeutic EVs applications [74–76]. The efficient and high-purity isolation of EVs from complex biological samples is the primary technical prerequisite for their in-depth research and clinical translation. Currently employed methods each possess distinct strengths: ultracentrifugation, the classical ‘gold standard’ relying on size and density differences for separation, is time-consuming and may cause mechanical damage to vesicles. size-exclusion chromatography better preserves the biological integrity and activity of EVs, yielding high purity. whereas strategies based on polymer precipitation or immuno affinity offer advantages in operational convenience and specificity respectively [72] (Fig. 2b). No single method is universally superior; rather, the choice necessitates a careful balance and optimization between yield, purity, activity preservation, and the specific objectives of downstream applications.

#### Engineering modification and activity enhancement strategies for EVs

EVs, as natural drug carriers, face challenges in clinical translation including insufficient targeting, low payload efficiency, and short circulation time in vivo. To overcome these bottlenecks, researchers have developed systematic engineering strategies aimed at enhancing their targeting capability, stability, and therapeutic efficacy. These approaches can be broadly categorised into endogenous modification and exogenous modification. Their core methodologies, advantages, and comparative analysis are summarised in the table below (Table 3).

**Table 3:** Overview of primary engineering strategies for EVs

Strategy	Principle	Key Advantages	Limitations	Ref.
Co-culture of drugs with target cells	Small molecules passively diffuse into cells and become encapsulated during EVs biogenesis	Simple operation, does not disrupt the natural structure of EVs	Low loading efficiency, dependent on cellular uptake capacity	[77–79]
Nucleic acid transfection	Genetic material is introduced into secretory cells, integrating its products into EVs	Enable stable genetic modification, suitable for nucleic acid therapeutics	Efficiency varies by cell type and vector, with risks of gene transfer	[80–82]
Surface protein engineering	Genetically edited secretory cells express membrane-targeted fusion proteins	Enable highly specific targeting or real-time imaging with precise modification	Process is time-consuming and only applicable to cell culture systems	[83, 84]
Co-culture of drugs with EVs	Loading relies on concentration gradients or electrostatic interactions	Mild conditions, minimal damage to EVs	Generally low efficiency, limited to specific molecular types	[85, 86]
Ultrasonic treatment	Acoustic cavitation temporarily disrupts membrane structures, forming pores	High loading efficiency, particularly suitable for macromolecules	May cause EVs aggregation or structural damage	[87]
Electroporation	Electrical pulses create reversible membrane pores, facilitating entry of charged molecules	Suitable for various charged macromolecules, with mature technology	May cause molecular aggregation and damage to EVs	[88, 89]
Extrusion method	Mechanical force propels EVs through filter membranes, achieving fusion and encapsulation	Formulations with uniform particle size can be obtained	May cause damage to membrane proteins and structures	[90, 91]
Freeze-thaw cycles	Ice crystal formation and melting modulate membrane permeability	Simple operation, no specialised equipment required	Moderate efficiency, may affect EVs stability	[90–92]
Saponification treatment	Saponins interact with membrane cholesterol, reversibly enhancing permeability	Mild conditions required to preserve EVs morphology	Residual saponins must be removed. haemolysis risk present	[90, 93]
Targeted peptide conjugation	Targeted ligands are immobilised on membranes via click chemistry or bio-linking	Significantly enhance tissue/cell-specific targeting	Conjugation chemistry may affect the intrinsic surface properties of EVs	[94–96]
PEGylation	Covalent attachment of polyethylene glycol forms a hydrophilic ‘invisible’ coating	Significantly prolong the in vivo circulation half-life and reduce clearance	May mask natural targeting signals and possess potential immunogenicity	[97–99]
Glycosylation	Metabolic labelling or enzymatic modification alters surface glycan structures	Regulate the interaction between EVs and receptors, influencing biodistribution	Technically complex, with in vivo effects potentially difficult to predict	[100–102]

The engineering of EVs constitutes a multi-tiered, modular integrated system. Endogenous strategies excel at achieving stable, genetically encoded functional modifications, yet remain constrained by cell sources and production cycles. Exogenous loading offers flexible, efficient payload integration solutions, though requiring meticulous optimisation to balance efficiency with membrane integrity. surface functionalisation, meanwhile, is pivotal for precisely regulating EVs behaviour in vivo (such as targeting and prolonged circulation). Moving forward, the intelligent combination of these strategies, tailored to specific therapeutic scenarios based on factors such as target type, payload properties, and delivery route, will become the core direction for developing next-generation, highly efficient, and intelligent EVs delivery systems.

#### EVs heterogeneity, dose standardisation and structure-function framework: current challenges and future directions

Research into the application of EVs in chronic wound treatment has advanced rapidly, yet it generally lacks systematic ‘dose-response’ and ‘structure-function’ analytical frameworks, constituting a core obstacle to its clinical translation [103–105]. The challenges manifest primarily across three interrelated dimensions: the inherent heterogeneity of EVs, the ambiguity and lack of standardisation in dosimetry, and the inadequacy of bioefficacy assessment systems [106].

Firstly, the therapeutic efficacy of EVs is highly dependent on their heterogeneity, which is determined by their source, preparation process, and engineering strategies. EVs derived from different cellular sources carry unique molecular payloads, thereby exhibiting specific functional tendencies. For instance, mesenchymal stem cell-derived EVs (MSC-EVs) excel in immunomodulation and pro-angiogenesis [107–109], whilst those derived from M2 macrophages or platelets respectively emphasise anti-inflammatory and repair-initiating functions [109]. Plant-derived EVs have also garnered attention for carrying distinct bioactive molecules [110]. However, even homologous EVs exhibit variations in size, membrane composition, and cargo due to differing cell culture conditions (e.g., hypoxic preconditioning [111, 112]) and isolation methods [113, 114]. Most studies employ only basic characterisation (e.g., electron microscopy morphology, particle size, and marker proteins CD9/CD63 [115, 116]), lacking detailed analysis of subpopulations and deeper molecular composition, resulting in poor product comparability [117, 118]. Further engineered modifications (such as loading specific miRNAs [119, 120] or performing membrane surface modifications [121, 122]) enhance functionality but significantly increase product complexity and quality control challenges [27, 123, 124].

Secondly, the confusion surrounding dose reporting and the ambiguity of dose-response relationships severely impede the optimisation and comparison of treatment regimens. Current literature exhibits extreme inconsistency in EVs dosage units, commonly reported as particle counts (e.g., 10¹⁰ particles [125, 126], protein mass (e.g., 100 µg [109, 127]), or vague volumetric descriptions, rendering the establishment of a universal effective dosage range virtually impossible [27, 128]. The vast majority of studies employ only a single empirical dose for experimentation [104, 105], lacking systematic gradient dose studies to elucidate dose-response relationships, minimum effective doses, or potential toxicity [103, 129]. When EVs are loaded into delivery systems such as hydrogels for sustained release [130, 131], the interplay between total loading capacity, encapsulation efficiency, and release kinetics further complicates the determination of locally effective doses [132, 133], underscoring the urgency of establishing a precise dosimetric framework.

Finally, a “structure-function” framework linking the physicochemical properties of EVs to their biological functions remains unestablished, and standardised efficacy assessment methods are lacking. Our understanding of how EVs size and membrane protein composition influence their penetration into wound matrices and uptake efficiency by target cells remains limited. Although numerous studies have identified active molecules within EVs through omics approaches (e.g., miR-494-3p [134]) and validated associated signalling pathways (e.g., PI3K/Akt [120], HIF-1α [115]), these findings predominantly demonstrate correlations rather than definitive causal evidence. More critically, current efficacy assessment methods relying on downstream phenotypes (e.g., cell migration, angiogenesis) remain inconsistent and difficult to quantify. This inability to establish standardised efficacy evaluation for predicting in vivo therapeutic outcomes constitutes a major bottleneck for regulatory approval and clinical translation [27, 128].

In summary, advancing EVs towards clinical application necessitates future efforts in: (1) Promoting standardisation by adopting dual metrics of particle count and protein mass in research reports, whilst adhering to guidelines such as MISEV for characterisation and production [135, 136]. (2) Deepening mechanism studies to systematically explore quantitative relationships between key EVs attributes and their in vivo distribution and cellular interactions. (3) Establishing clear preclinical dose-response curves. (4) Industry-academia-research collaboration to develop standardised in vitro efficacy assays correlated with in vivo therapeutic outcomes [27, 137]. Only by systematically addressing these challenges can EVs be transformed from a promising yet heterogeneous ‘bioactive mixture’ into the next generation of biologic therapeutics with controllable quality and predictable efficacy [132, 138].

### The Construction of EVH

#### Synthesis methods of EVH

**Fig. 3:**
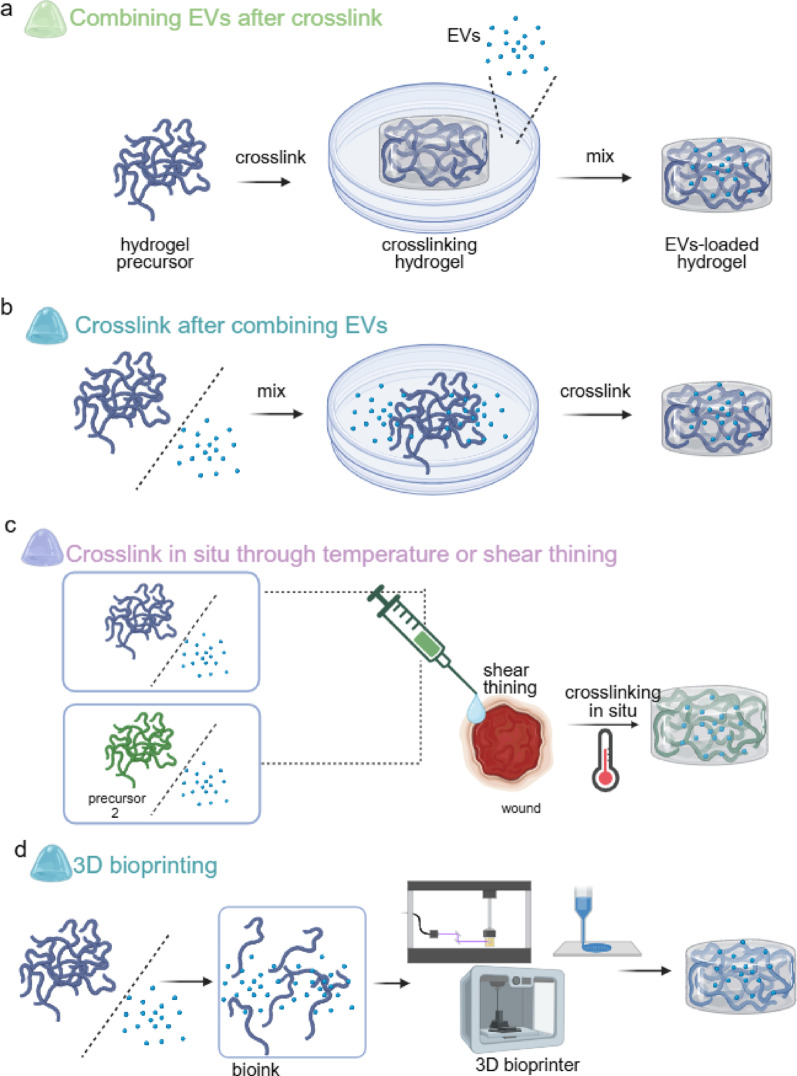
The synthesis methods of EVs combined with hydrogels for wound healing. The synthetic approaches for EVs-loaded hydrogels can be categorized as follows: (**a**) Combining EVs after crosslink: sequential crosslinking followed by EVs absorption. (**b**) Crosslink after combining EVs: pre-mixing EVs with hydrogel precursor and subsequent crosslinking. (**c**) Crosslink in situ through temperature or shear thining : simultaneous injection of cross-linkable precursors mixed with EVs at the wound site for in-situ gelation. (**d**) 3D bioprinting: formation of a bio-ink by combining EVs and hydrogel precursors, followed by 3D printing technology for composite hydrogel preparation(Figure created using BioRender) https://BioRender.com

Based on the timing and mechanism of combining EVs with hydrogels, current common loading strategies can be categorised into four types: post-loading, pre-gel encapsulation, in situ cross-linking, and 3D bioprinting technology **(**Fig. 3). These methods each exhibit distinct characteristics regarding EVs distribution uniformity, loading efficiency, release kinetics, and preservation of EVs integrity, necessitating selection according to specific application scenarios. Detailed descriptions of each approach follow.

##### Combining after crosslink

The post-loading method, also termed the “breathing technique”, involves first cross-linking the hydrogel precursor to form a network structure, followed by physically adsorbing EVs onto the preformed hydrogel **(**Fig. 3a). The standard procedure entails immersing dehydrated or pre-swelled hydrogels in an EVs-containing solution, where EVs diffusion and retention within the pores occurs via hydrogel network swelling and capillary action. This method is straightforward to implement and avoids exposing EVs to potentially harmful cross-linking conditions (such as ultraviolet light or reactive chemical cross-linking agents). However, its loading efficiency and uniformity are highly dependent on the match between the hydrogel pore size and the EVs particle size. If the pores are too large, rapid burst release of EVs may occur. If the pores are too small, EVs struggle to enter effectively, limiting the drug loading capacity. For instance, this absorption-based loading approach was employed in preparing egg white hydrogel-loaded lyophilised EVs (EWG@L-EVs) [139]. This method is particularly suitable for EVs systems sensitive to cross-linking conditions, where chemical or physical stimulation must be avoided.

##### Crosslink after combining EVs

The pre-gel encapsulation method involves uniformly mixing EVs with a liquid hydrogel precursor solution prior to the hydrogel’s cross-linking reaction **(**Fig. 3b). Subsequently, gelation is triggered through physical means (such as temperature changes) or chemical methods (such as photoinitiation or ionic cross-linking), encapsulating the EVs within the resulting three-dimensional network. This approach enables uniform distribution of EVs within the gel and precise control over loading quantities [140, 141]. The key lies in selecting mild cross-linking conditions to maximise EVs integrity preservation. For instance, mixing adipose-derived stem cell exosomes with a polymer solution followed by dynamic borate ester cross-linking under mild conditions yields multifunctional hydrogels [17]. Calcium alginate hydrogels utilising ionic cross-linking frequently employ this approach, achieving rapid gelation and encapsulation of EVs in the presence of calcium chloride. This method offers advantages including high encapsulation efficiency and uniform distribution, though careful optimisation of cross-linking parameters is essential to prevent EVs damage.

##### Crosslink in situ through temperature or shear thining

The in situ cross-linking method involves directly applying a mixture of EVs and a flowable hydrogel precursor to the wound surface **(**Fig. 3c). Under physiological conditions such as body temperature, physiological pH, or contact with tissue fluid, the mixture forms a gel directly at the wound site. This strategy is particularly suited to irregular or deep wounds, enabling complete filling of the wound cavity and local site-specific delivery of EVs. Thermosensitive hydrogels (e.g., Pluronic F127) exemplify this approach. Liquid at low temperatures, they rapidly gelify upon injection into the wound at body temperature, enabling in situ encapsulation and sustained release of EVs [142, 143].An alternative approach employs separately stored two-component precursors (e.g., hyaluronic acid modified with hydrazide and aldehyde groups respectively). These are simultaneously ejected onto the wound using a dual-chamber syringe, where they undergo immediate Schiff base reaction cross-linking to form a gel [144]. This method offers minimally invasive and conformable advantages, though it necessitates ensuring precursor biocompatibility alongside a rapid and stable gelation process.

##### 3D Bioprinting

3D bioprinting technology employs computer-aided design to sequentially deposit hydrogel ‘bio-inks’ containing EVs, constructing personalised scaffolds with intricate three-dimensional structures and predefined porosity **(**Fig. 3d). This technique provides an unprecedented platform for the temporally and spatially controlled release of EVs and the simulation of tissue-specific microenvironments. For instance, bio-inks combining GelMA and HA-DA can be employed to print dual-layer network scaffolds promoting cartilage regeneration, enabling targeted EVs release [145]. Integrating freeze-dried EVs preparations (Lyosecretome) with sodium alginate-sericin (SA-SF) hybrid bio-inks for 3D printing allows precise regulation of EVs release kinetics [146, 147].Compared to conventional physical mixing or adsorption methods, 3D-printed scaffolds typically exhibit superior EVs controlled-release efficacy and enhanced tissue integration capacity [148]. However, this technique imposes specific requirements on the rheological properties of the bioink (e.g., viscosity, shear-thinning behaviour) and the printing process (e.g., extrusion pressure, UV irradiation), necessitating meticulous optimisation to prevent compromise of EVs activity.

In summary, the selection of loading strategies should be based on a comprehensive assessment of the characteristics of the target wound surface, the required EVs release kinetics, the source and sensitivity of the EVs, and the actual clinical operating conditions.

#### Controlled release mechanisms of EVs from EVH system

The release behaviour of the EVH system constitutes its core kinetic characteristic for exerting therapeutic effects. An ideal EVH system should achieve sustained, controllable, and responsive release of EVs to align with the dynamic stages of wound healing (inflammatory, proliferative, and remodelling phases), whilst preventing loss of bioactivity or local under-dosing due to burst release phenomena. This chapter systematically analyses the release kinetics mechanisms of action and influencing factors of various EVH systems based on literature data, aiming to provide theoretical foundations for the rational design of highly efficient EVH systems **(**Table 4**)**.

##### Release mechanism

Diffusion-dominated release represents the most fundamental and prevalent passive delivery mechanism, whereby EVs diffuse into the external environment through pores or aquapores within the hydrogel network. The release rate is primarily regulated by the hydrogel’s crosslinking density, porosity, swelling ratio, and non-specific interactions between EVs and the matrix (such as electrostatic and hydrophobic forces). For instance, adjusting the substitution degree of methacrylamidated gelatin (GelMA) modifies the network crosslinking density, thereby controlling the diffusion rate of EVs [115, 119, 144]. Studies indicate that a relatively loose network structure coupled with moderate swelling promotes steady and sustained diffusion of EVs.

To achieve intelligent, on-demand release, researchers have developed multiple stimulus-responsive delivery strategies. These primarily utilise biochemical signals from the wound microenvironment or external physical stimuli to trigger release, specifically including: Microenvironment-responsive degradation and dissociation: enzymatic degradation of hydrogel matrices (e.g., MMP-sensitive peptides [149]), hydrolysis, or dynamic covalent bond cleavage (e.g., pH-responsive hydrazone bonds, imine bonds [150, 151], ROS-responsive borate bonds, thioketone bonds [152, 153], can actively accelerate network degradation or dissociation under specific pathological conditions of diabetic wounds (e.g., elevated MMP, acidic pH, high ROS, and hyperglycaemia), thereby promoting EVs release. 2) Affinity interaction regulation: Engineering strategies enhancing EVs-matrix interactions—such as utilising tannic acid’s phenolic hydroxyl groups [154] or metal-organic framework materials [155] for enrichment and controlled release or chemically coupling EVs with oxidised hyaluronic acid [156]—enable slower, more sustained release kinetics. 3) External Stimulus-Triggered Release: Incorporating thermosensitive materials (e.g., Pluronic F-127) enables body temperature-induced in situ gelation and sustained release [157, 158]. whereas near-infrared light-responsive systems incorporating photothermal agents (e.g., polydopamine, black phosphorus) can induce gel phase transitions or relaxation via photothermal effects, enabling on-demand release of EVs [159]. Collectively, these intelligent strategies achieve precise alignment between EVs release and the dynamic processes of wound healing [160].

##### Analysis of key influencing factors

Natural polymers (such as gelatin/GelMA, collagen, alginate, chitosan, hyaluronic acid) are widely utilised due to their excellent biocompatibility [161], though their degradation and release behaviour may exhibit batch-to-batch variability. Synthetic or semi-synthetic polymers (e.g., PEG, Pluronic F-127) possess more defined structures and predictable phase transition/degradation kinetics, facilitating controlled release [119]. Intelligent structural designs, such as core-shell microneedles [162], bilayer scaffolds [163], and nanofibre/particle composite hydrogels [164], enable more complex time-controlled or stimulus-responsive release through spatial compartmentalisation or secondary controlled-release units.

EVs derived from different cellular sources exhibit variations in size, membrane composition, and surface charge, which directly influence their diffusion behaviour and retention time within hydrogel networks. Engineered modified exosomes (e.g., overexpressing specific miRNAs [119]) or pretreated EVs (e.g., hypoxic preconditioning, references [165]) may exhibit no intrinsic alterations in release kinetics, yet their therapeutic efficacy demonstrates enhanced ‘timeliness’ due to functional augmentation. Loading methods have evolved from simple physical mixing (the mainstream approach) to more advanced strategies, such as pre-encapsulating EVs within microspheres [166], nanoparticles [167], or self-assembled clusters [111], followed by incorporation into gels to form secondary controlled-release systems. These methods more effectively prevent EVs inactivation and significantly prolong release duration.

The release data from most studies remain at a qualitative or semi-quantitative level, lacking standardised in vitro release kinetic curves (such as cumulative percentage released versus time plots) and key parameters (such as release half-life and rate constants). This hinders direct quantitative comparisons between different systems and optimisation of designs. Future research should focus on establishing unified release characterisation standards and delving into the quantitative relationship between ‘release kinetics-cell uptake efficiency-in vivo efficacy’. This will propel EVH systems towards more precise, efficient, and clinically translatable development.

**Table 4:** Summary of representative EVH system release characteristics

Hydrogel Type	EVs Source	Release Profile	Release Mechanism	Ref.
Thermosensitive (Pluronic F127)	ADSCs	Sustained release over 14 days, with an initial burst phase	Diffusion combined with hydrogel erosion	[142]
Photocrosslinked (GelMA)	HUCMSCs	~ 70% cumulative release within 7 days, fitting the Higuchi model	Synergistic diffusion and network degradation	[168]
Dynamic Covalent Bond (Boronate Ester)	ADSCs	Rapid release (~ 80% within 2 h) triggered by H₂O₂ or glucose	Dual-responsive to ROS and glucose	[17]
Enzyme-responsive (MMP-9-cleavable PEG)	ADSCs	Accelerated release (approximately 2.5-fold) in the presence of MMP-9	MMP-9 enzymatic cleavage	[63]
Ionically Crosslinked (Calcium Alginate)	ADSCs	Sustained release over 10 days, reaching ~ 60% cumulative release	Predominantly diffusion-controlled, with minor matrix dissolution	[169]
3D Bioprinted (SA/SF)	MSC-Lyosecretome	More sustained and prolonged release, extending beyond 28 days	Diffusion path regulated by the printed architecture	[146, 147]
Self-healing Injectable (CS-based)	BMSCs	Sustained release over 7 days, supporting cell migration	Diffusion through a dynamically bonded network	[170]

#### Advantages of the EVH System Over Alternative Therapeutic Approaches

Chronic wound healing impairment involves the interplay of multiple pathological pathways, rendering monotherapy frequently ineffective. The EVH system achieves functional synergy and complementarity by integrating materials engineering with cell biology. Its pronounced therapeutic efficacy stems from systematic optimisation of delivery efficiency, biological activity, and the wound microenvironment.

Free EVs are susceptible to enzymatic degradation at wound sites, exhibit short residence times, and present challenges in controlling release kinetics. Hydrogels, by establishing a localised “active reservoir”, not only provide EVs with a physical barrier against adverse microenvironments but also modulate release kinetics through their network architecture [156, 171]. For instance, hydrogels engineered with dynamic covalent bonds (e.g., Schiff base, borate ester) dissociate in response to wound-specific high ROS, low pH, or specific enzymes (e.g., matrix metalloproteinases), enabling on-demand, precise EVs release [149–151]. Moreover, the injectability, self-healing properties, and strong tissue adhesion of hydrogels (e.g., dopamine-modified GelMA) ensure close conforming to irregular wound surfaces, significantly enhancing the retention and utilisation efficiency of EVs at the lesion site [107, 172].

The therapeutic potential of EVs themselves has been specifically enhanced through engineered strategies. Hydrogels address the “delivery” challenge, whilst the core efficacy lies in the bioactive substances carried by EVs. Functionally enhanced EVs can be obtained by screening specific cell sources or pre-treating parental cells. For instance, EVs derived from hypoxic-pretreated mesenchymal stem cells or M2 macrophages exhibit enriched pro-angiogenic and anti-inflammatory factors within their contents, demonstrating superior reparative capabilities in animal models [112, 173, 174]. Genetic engineering of progenitor cells enables the production of engineered EVs carrying specific therapeutic molecules (e.g., miR-221-3p, miR-17-5p). This transforms them from natural messengers into ‘smart drugs’ capable of precisely regulating target pathways (e.g., the PTEN/p21 pathway), thereby addressing critical issues such as impaired angiogenesis [119].

The combination of hydrogels and EVs generates multidimensional synergistic effects, transcending simple physical mixing to achieve simultaneous intervention across multiple pathological pathways. In immune regulation, the sustained release of MSC-derived EVs or M2 macrophage-derived EVs from the hydrogel effectively guides macrophage polarisation towards the anti-inflammatory, repair-promoting M2 phenotype [175, 176]. When the hydrogel itself incorporates anti-inflammatory components (such as chitosan), a synergistic effect occurs, jointly suppressing excessive inflammation [177]. Regarding tissue regeneration promotion, the hydrogel’s three-dimensional structure provides a physical scaffold for cellular migration and proliferation. Concurrently, the sustained release of EVs continuously supplies signals that promote angiogenesis (e.g., by activating the HIF-1α/VEGFA pathway [115]) and cell migration. This dual action synergistically accelerates granulation tissue formation, neovascularisation, and epithelial coverage [178].

Currently, this field is advancing towards the development of integrated, multifunctional systems. Cutting-edge research no longer settles for binary combinations but focuses on creating intelligent platforms that integrate multiple therapeutic modalities. For instance, combining EVH systems with photothermal conversion nanomaterials enables responsive release of EVs while providing on-demand photothermal antibacterial capabilities [179]. Alternatively, composites with oxygen carriers (e.g., haemoglobin) or oxygen-generating materials (e.g., MnO₂) enhance EVs’ pro-angiogenic effects while alleviating tissue hypoxia [144, 159]. These designs aim to simultaneously address the intertwined challenges of infection, ischaemia, inflammation, and cellular dysfunction in diabetic wounds through a single integrated dressing, representing a novel paradigm for chronic wound therapy.

## Applications for EVH systems

### ADSCs-derived EVH systems for wound healing

#### Overview of ADSCs-EVH

**Fig. 4:**
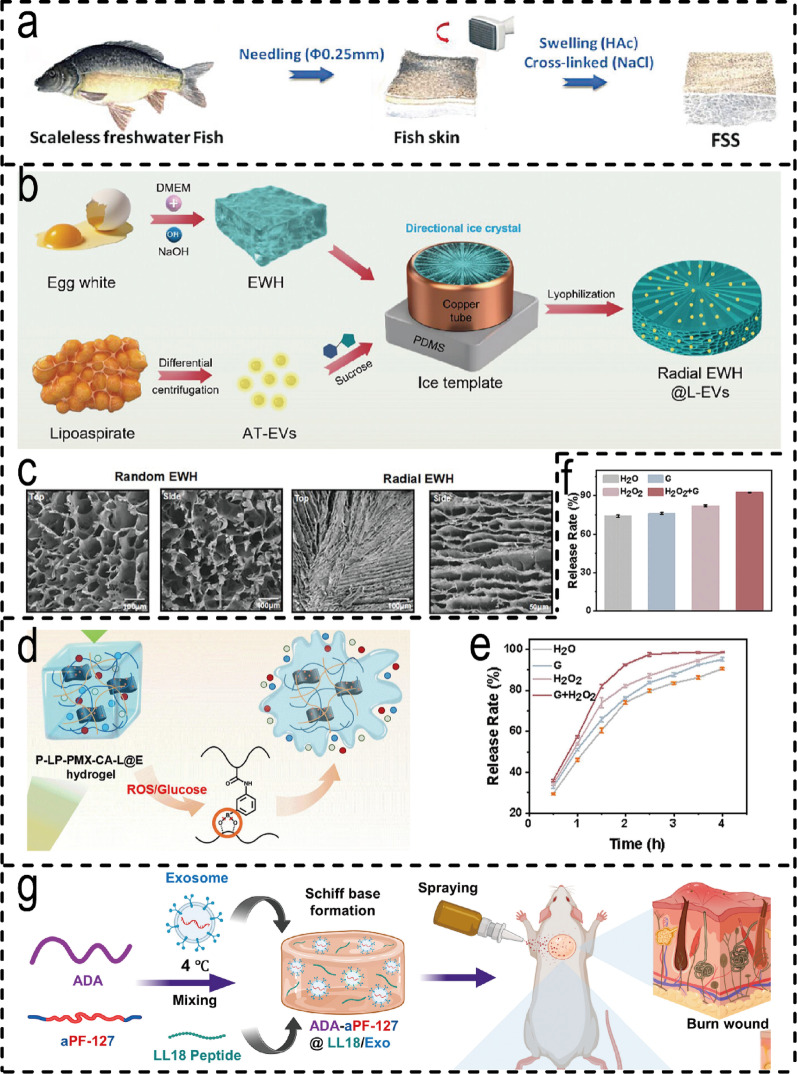
Types of hydrogels for ADSCs-Derived EVH systems. (**a**) The FSS prepared from scaleless freshwater fish skin treated with acetic acid (HAc) and cross-linked with NaCl has a suitable structure for exosomes loading [180]. (Copyright 2023, John Wiley and Sons.) (**b**) Schematic of radial EWH@L-EVs preparation. (**c**) SEM image presented a plane view of the top and side parts of EWH with different structures. Scale bar = 100 μm [139]. (Copyright 2024, Elsevier.) (**d**) ROS/glucose-responsive chemical structure in hydrogels. (**e**) ROS/glucose response of P-LP-PMX-CA-L hydrogel. (**f**) Chlorogenic acid release rate of P-LP-PMX-CA-L hydrogel after 2 h exposure to H_2_O_2_/glucose system [17]. (Copyright 2024, Elsevier.) (**g**) Schematic diagram showing the fabrication of ADA-aPF127@LL18/Exo for burn wound healing [181]. (Copyright 2024, Elsevier.)

ADSCs-EVs possess core functions including promoting angiogenesis, exhibiting anti-inflammatory properties, and supporting multi-lineage differentiation [17, 63]. To maximise their therapeutic efficacy, it is necessary to select a compatible hydrogel delivery platform. These platforms can be categorised into three primary types: firstly, natural hydrogels that provide a biomimetic microenvironment, such as fish skin collagen scaffolds (FSS) [180] (Fig. 4a) which facilitate cell infiltration and EVs loading, and radial egg white hydrogels (EWH) [139] capable of directing EVs release to guide cell migration(Fig. 4b, c). Secondly, synthetic/composite hydrogels capable of controlled release, such as GelMA [110] and thermosensitive Pluronic F127 [142] hydrogels that precisely regulate mechanical properties and degradation behaviour; Thirdly, intelligent responsive hydrogels capable of addressing complex pathologies, such as PEG hydrogels responding to matrix metalloproteinases (MMPs) releasing EVs in diabetic wounds [63], dual ROS/glucose-responsive hydrogels dynamically alleviating oxidative stress and inflammation [17] (Fig. 4d-f), and sprayable thermosensitive polysaccharide hydrogels alleviating hypoxia while conforming to wound surfaces [181] (Fig. 4g) and oxygen-releasing cryogels [182].

#### Molecular mechanisms of ADSCs-EVH in promoting wound healing

**Fig. 5:**
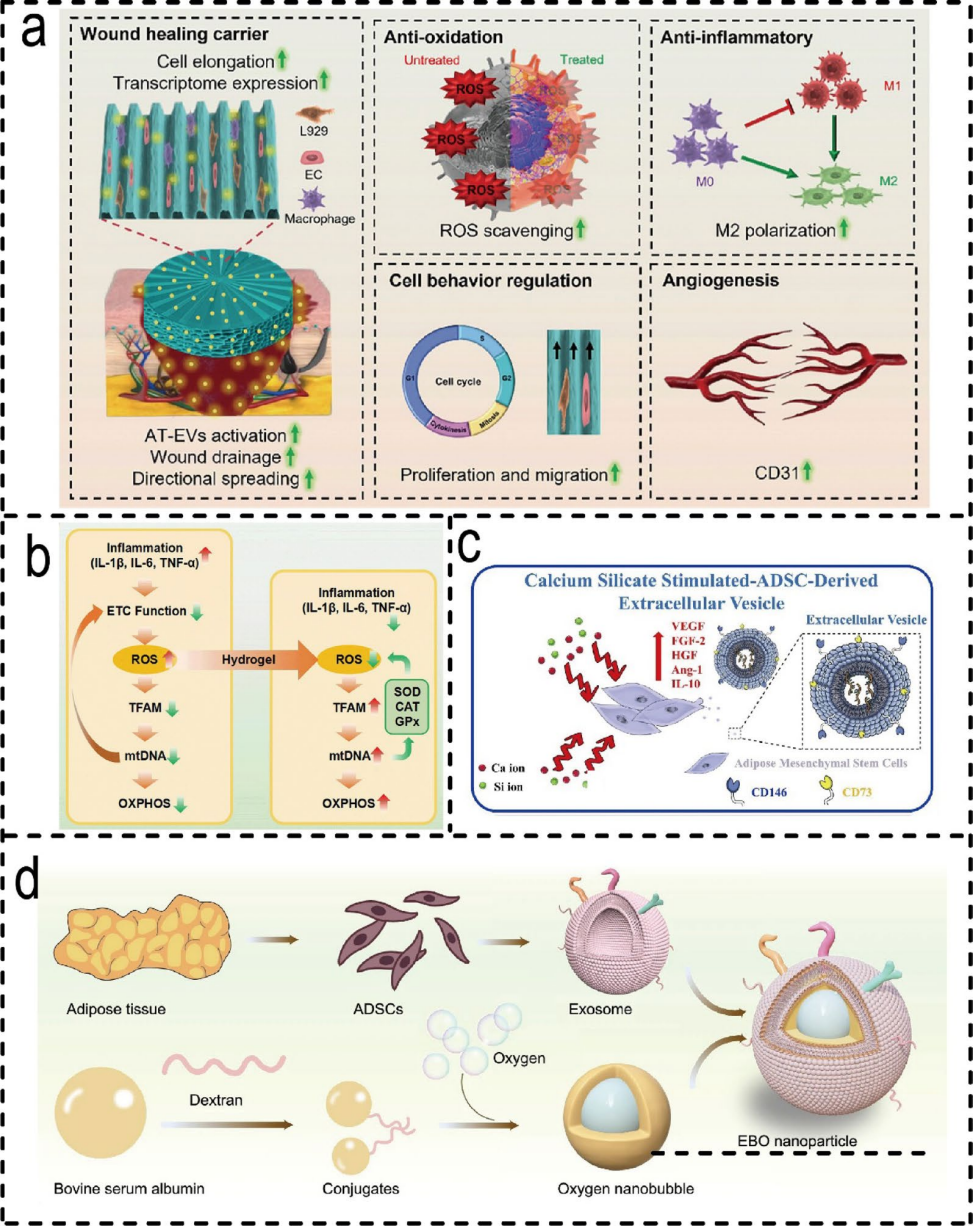
Mechanism of action of ADSCs-EVH systems and modification of EVs. (**a**) Orchestrating Healing Phases: Spatiotemporal Cargo Release and Multidimensional Fate Guidance to Accelerate Diabetic Skin Regeneration [139]. (Copyright 2024, Elsevier.) (**b**) Potential mechanism of action [17]. (Copyright 2024, Elsevier.) (**c**) the stimulation of ADSCs by calcium (Ca) and silicon (Si) ions to produce EVs enriched with growth factors and anti-inflammatory cytokines [186]. (Copyright 2025, Springer Nature) (**d**) Schematic of the preparation of ONB and EBO [185]. (Copyright 2024, Springer Nature.)

The healing efficacy of the ADSC-EVH system stems from its multifaceted regulation of key signalling pathways, oxidative stress, and mitochondrial function. For instance, EVs activate pathways such as Wnt/β-catenin to promote angiogenesis [131], whilst reducing ROS levels by approximately 50% through mitochondrial modulation [183] (Fig. 5b). Engineering strategies to enhance EV functionality synergistically with hydrogel smart responsiveness achieve synergistic therapeutic effects: hypoxic pre-conditioning of ADSC-EVs amplifies their pro-angiogenic capacity [184]; composite hydrogel systems integrating EVs with oxygen nano-bubbles simultaneously alleviate hypoxia and facilitate intracellular delivery [185] (Fig. 5d). while the spatial control provided by radial EWH hydrogels synergises with EVs functions to effectively guide cellular behaviour [139] (Fig. 5a). Calcium silicate-stimulated ADSC-EVs promote ordered collagen arrangement and epithelialisation [186] (Fig. 5c).

### HUVECs-Derived EVH systems for wound healing

#### Overview of HUVECs-EVH

**Fig. 6:**
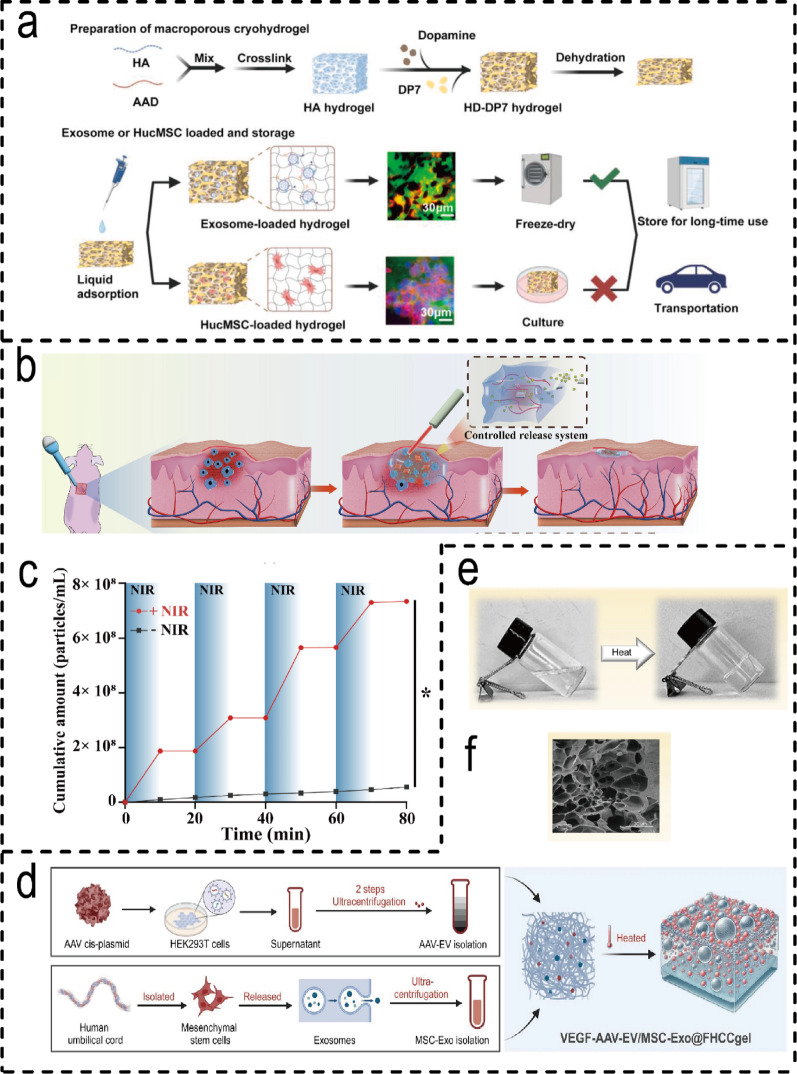
Types of hydrogels for HUVECs-Derived EVH systems. (**a**) Schematic of the preparation of polydopamine and antimicrobial peptide DP7 coated hyaluronic acid hydrogel. The prepared HD-DP7 hydrogel can be loaded with HucMSCs or HucMSC-Exos using the solution adsorption method. HD-DP7/Exo can be lyophilized for long-term storage or long-distance transport, while HD-DP7/HucMSC can only be cultured for short-term use [187]. (Copyright 2024, Elsevier.) (**b**) Schematic illustration of novel exosome-based multifunctional nanocomposite platform driven by photothermal-controlled release system for repair of skin injury. (**c**) Release kinetics of hucMSC-derived exosome from hucMSC-derived exosome/Ti3C2 MXene hydrogel with and without NIR laser irradiation [189]. (Copyright 2024, Elsevier.) (**d**) VEGF-EV-AAV/MSC-Exo@FHCCgel construction. (**e**) Visual confirmation of the temperature-induced gelation of the hydrogel system. (**f**) SEM representation of the VEGF-EV-AAV/MSC-Exo@FHCCgel structure [190]. (Copyright 2025, Springer Nature.)

EVs derived from HUVECs possess core bioactive properties that promote angiogenesis and are widely selected for wound healing therapies. Natural polymer hydrogels (e.g., hyaluronic acid, chitosan) are favoured for their inherent biological affinity. For instance, freeze-dried hyaluronic acid hydrogel sustainably preserves exosome activity over extended periods (Fig. 6a) [187], whilst chitosan-based hydrogels effectively combat infection and promote vascularised granulation tissue formation [188]. Smart-responsive hydrogels further advance precision delivery strategies: photothermally responsive Ti₃C₂ MXene/agarose systems enable near-infrared laser-controlled on-demand release (Fig. 6b, c) [189]. Thermosensitive VEGF-EV-AAV/MSC-Exo@FHCCgel undergoes sol-gel transition at physiological temperatures (Fig. 6d-f), enabling sustained controlled vesicle release [190]. Synthetic hydrogels (e.g., GelMA) offer tunable physicochemical properties and sustained-release capabilities [168]. Consequently, for targeted delivery of specific functional vesicles such as HUVECs-EVs, hydrogel platform design must precisely align with their biological characteristics and the stage-specific demands of wound repair, enabling synergistic therapy spanning angiogenesis to tissue remodelling.

#### Molecular mechanisms of HUVECs-EVH in promoting wound healing

**Fig. 7:**
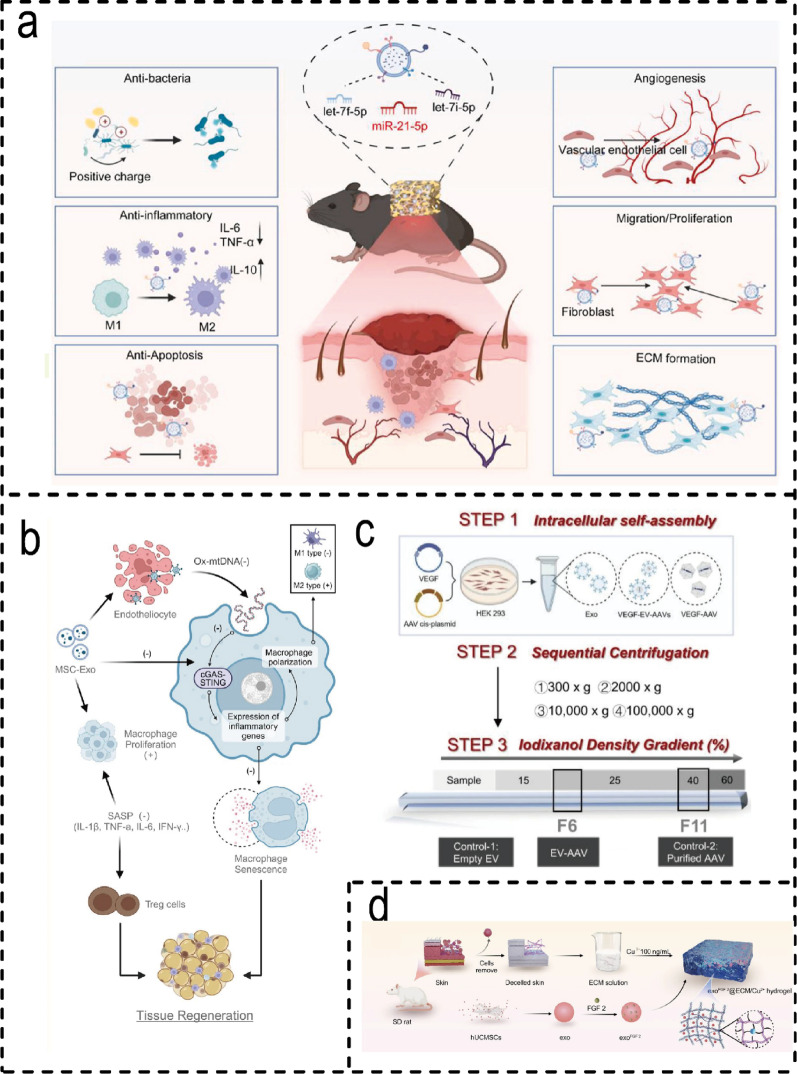
Mechanism of action of the HUVECs-EVH systems and modification of EVs. (**a**) HD-DP7/Exo can promote scar-free healing of deep second-degree burn wounds with infection through antibacterial action, anti-apoptotic effects, anti-inflammatory properties, promotion of angiogenesis, enhancement of reepithelialization, and regulation of extracellular matrix synthesis [187]. (Copyright 2024, Elsevier.) (**b**) Schematic Diagram of MSC-EXO Promoting Macrophage M2 Polarization. (**c**) Synthesis of EV-AAV through iodixanol-based high-speed centrifugation [190] (Copyright 2025, Springer Nature.) (**d**) ECM solution was obtained from rat skin and a trace amount of Cu^2+^ was used as a cross-linking agent. exoFGF 2 were prepared by sonicating exosomes with FGF_2_ [193]. (Copyright 2024, Springer Nature.)

In recombinant human collagen hydrogels (MSC-exos@RHCMA), umbilical cord mesenchymal stem cell-derived exosomes (ucMSC-exos) demonstrated superior efficacy to adipose or bone marrow-derived exosomes in alleviating inflammation, promoting angiogenesis, and enhancing collagen formation [191]. whereas human umbilical cord blood or umbilical cord-derived mesenchymal stem cell-derived exosomes (e.g., CS-EVs hydrogel) achieved a 93.3% closure rate of diabetic ulcer wounds by promoting HUVEC endocytosis and upregulating angiogenesis markers [192]. Regarding immunomodulation, distinct systems exhibit unique characteristics: CB-Treg-exos/Pluronic F-127 hydrogels induce monocyte polarisation towards the anti-inflammatory M2 phenotype and increase the proportion of M2 macrophages in vivo [126]. CMCS/OHA/Exo hydrogels promote macrophage phenotypic conversion and suppress pro-inflammatory cytokines [176]; while freeze-dried HD-DP7/Exo hydrogels (Fig. 7a) coordinate regulation of fibroblasts, vascular endothelial cells, and macrophages, inhibiting myofibroblast-mediated fibrosis and promoting scar-free healing after burns [187]༛rhCol III-EVs hydrogels drive M1-to-M2 macrophage polarisation, synergistically enhancing epithelial regeneration and collagen synthesis [177]. Regarding functional optimisation, bioengineered exosomes (e.g., those enriched with mitochondrial oxidative phosphorylation proteins) outperform natural exosomes in accelerating fibroblast proliferation and wound repair [193]. Engineered systems also exhibit diverse advantages: for instance, systems combining adeno-associated viruses (VEGF-EV-AAV) (Fig. 7b, c) enhance gene delivery efficiency [190], while systems complexed with growth factors (exoFGF2@ECM/Cu²⁺) boost antimicrobial activity (Fig. 7d) [193].

### M2 Macrophage/ BMSCs-derived EVH systems for wound healing

#### Overview of M2 macrophage/ BMSCs-derived EVH

**Fig. 8:**
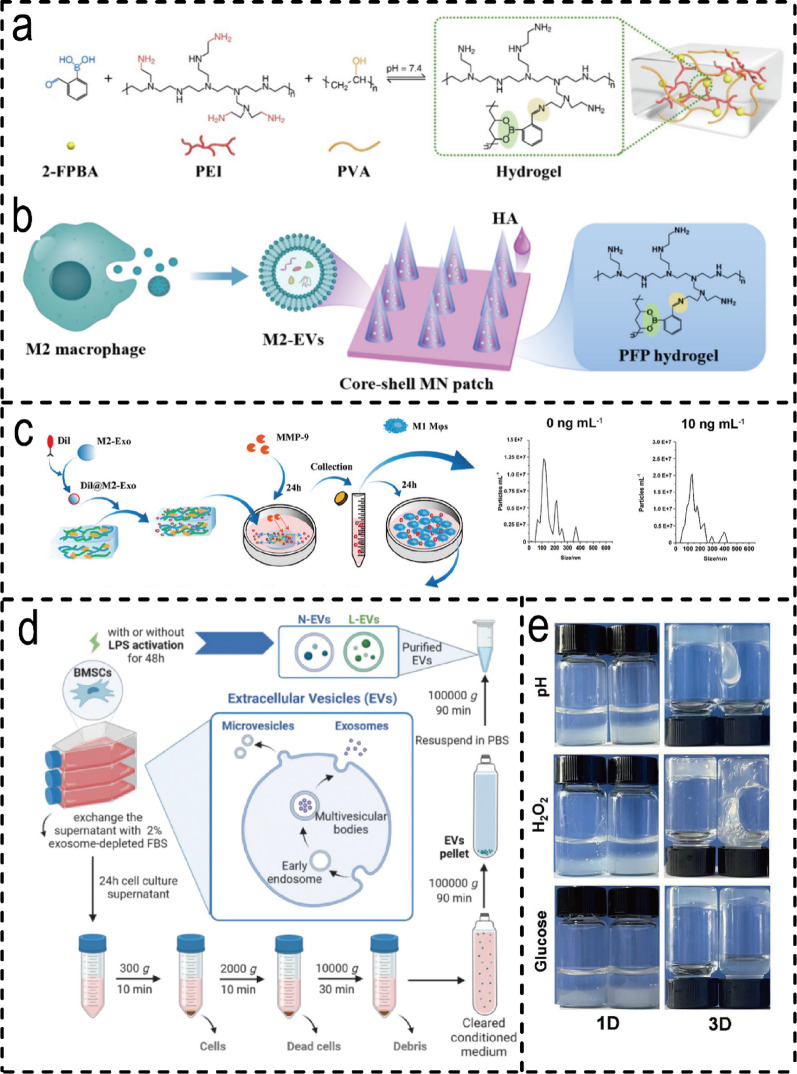
Types of hydrogels for M2 Macrophage/ BMSCs-derived EVH systems. (**a**) Schematic diagram of the preparation process for PVA/2-FPBA/PEI dual-crosslinked hydrogels. (**b**) Schematic illustration ROS-scavenging microneedles with M2 macrophage vesicles encapsulation [150]. (Copyright 2024, Elsevier.) (**c**) Schematic illustration of the in-vitro experiment process. and NTA results of exosomes in the supernatant of each group [149]. (Copyright 2025, John Wiley and Sons.) (**d**) Schematic diagram of EVs isolation and purification [197]. (Copyright 2024, John Wiley and Sons.) (**e**) Weight change of different hydrogels in different environments [18]. (Copyright 2023, Elsevier.)

Strategic design of hydrogel systems for delivering EVs derived from M2 macrophages or BMSCs primarily centres on three distinct categories: natural, synthetic, and smart responsive systems. Each exhibits unique characteristics and inherent trade-offs in efficacy. Natural hydrogels (e.g., GelMA, alginate) are widely adopted due to their excellent biocompatibility and tunable physical properties. Examples include GelMA hydrogels loaded with mimetic EVs [194] and self-healing CEC-DCMC hydrogels [104]. However, their limited mechanical strength predisposes them to premature degradation in dynamic wounds [195]. Synthetic polymer hydrogels (e.g. degradable PEG) allow for the precise regulation of crosslink density, enabling controlled degradation and sustained exosome release for up to 27 days [196]. However, synthetic materials may increase the risk of immunogenicity. Smart-responsive hydrogels represent a more advanced delivery paradigm, enabling on-demand EV release in response to specific signals from the wound microenvironment (e.g. MMP-9, ROS and glucose). MMP-9-responsive hydrogels can selectively release M2-Exos to accelerate the healing of diabetic wounds [149] (Fig. 8c), while ROS-clearing microneedle patches dissociate to release M2-EVs in the event of high oxidative stress [150] (Fig. 8a, b). Functionally optimised L-EVs, delivered via injectable sericin hydrogels, demonstrate superior repair outcomes in both wound and bone defect models [197] **(**Fig. 8d**)**. Similarly, glucose- and H₂O₂-responsive chitosan hydrogels adapt to fluctuations in the wound microenvironment for targeted exosome delivery **(**Fig. 8e**)**. Overall, the selection of hydrogel type requires careful balancing between biocompatibility, functional precision, and clinical translational feasibility to align with the specific delivery requirements of EVs in distinct therapeutic scenarios.

#### Molecular mechanisms of M2 Macrophage/ BMSCs-EVH in promoting wound healing

**Fig. 9:**
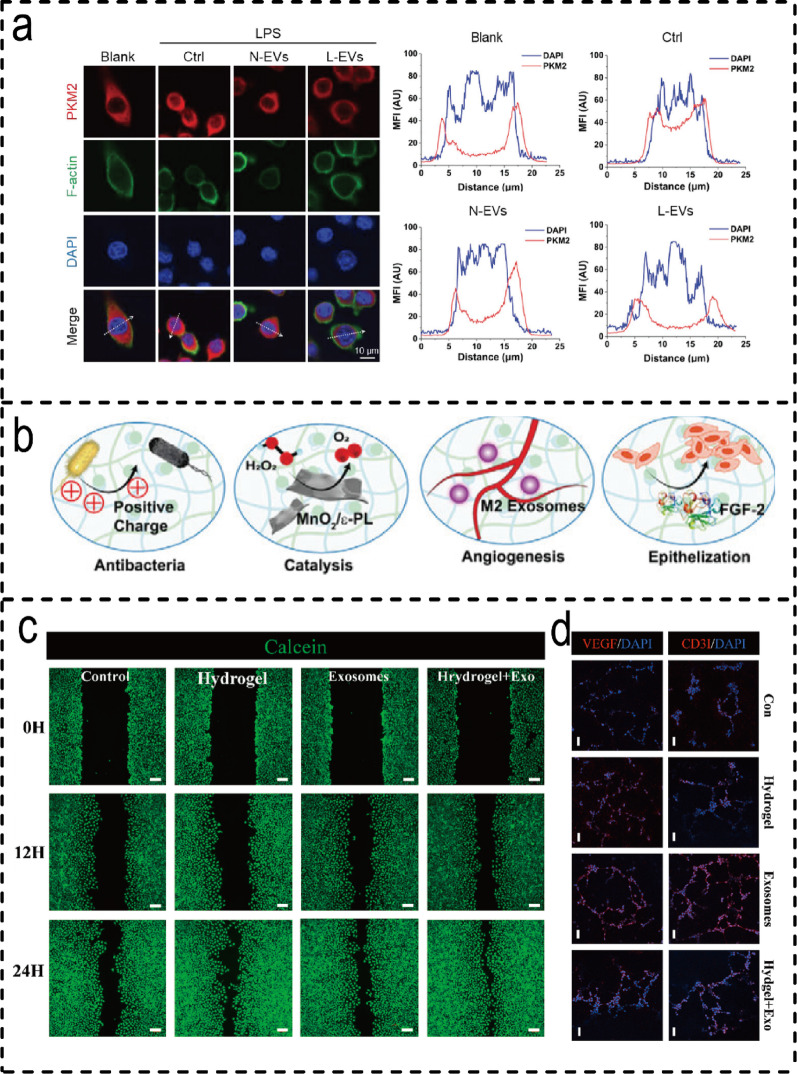
Mechanism of action of the M2 Macrophage/BMSCs-EVH systems. (**a**) Subcellular PKM2 localization was assessed via immunofluorescence staining and Line charts showing the mean fluorescence intensity (MFI) along the direction of the white dashed arrows [194]. (Copyright 2024, John Wiley and Sons.) (**b**) The positive quaternary ammonium groups in the graft play a long-term antibacterial role. The MnO_2_/e-PL nanosheet acted as a nanoenzyme to catalyze the conversion from H_2_O_2_ to O_2_. The released ExosM2@miR-223 simulated long-lasting angiogenesis and the released FGF-2 accelerated epithelization efficiently [196]. (Copyright 2023, John Wiley and Sons.) (**c**) Wound healing migration assay of HUVEC cells on each sample at different time points (*n* = 3). Scale bar: 200 μm (**d**) Immunofluorescence images of CD31 (red) and VEGF (red) in HUVEC cells forming vessels in each group (*n* = 3) [199]. (Copyright 2025, Elsevier.)

One of the core mechanisms by which EVs from this source exert their effects lies in promoting the polarisation shift of macrophages from the M1 to the M2 phenotype. For instance, Mo-Exo encapsulated in GelMA hydrogels enhances angiogenesis by activating the ERK1/2 pathway [194], whilst M2-Exos delivered via PEG hydrogels sustain phenotypic conversion for over six days to accelerate epithelial regeneration [196]. Regulatory pathways vary across systems: AG/NSPs hydrogels act via JAK/STAT and PPAR pathways [198], whilst L-EVs promote M2 maturation by inhibiting PKM2 nuclear translocation [197](Fig. 9a). Angiogenesis may be further enhanced through synergistic factor delivery, such as the HA@MnO₂/FGF-2/Exos hydrogel simultaneously targeting oxidative stress and tissue repair [199] (Fig. 9b). Microenvironment-responsive designs (e.g., MMP-9-responsive hydrogels [149] and ZIF-8 composite systems [26, 200] ) enhance therapeutic precision. Furthermore, both GelMA-dopamine hydrogels [201] **(**Fig. 9c) and TATA hydrogels [202] (Fig. 9d) effectively promote angiogenesis and microenvironmental repair. Nevertheless, existing systems face limitations: ROS scavenging strategies lack universal applicability [150], oxygen-releasing dressings encounter scalability challenges, and the exosome sources in some systems remain poorly documented [18]. Consequently, future development must seek a more optimal balance between mechanism specificity, functional integration, and clinical feasibility.

### Plant-derived EVH systems for wound healing

#### Overview of plants-EVH

**Fig. 10:**
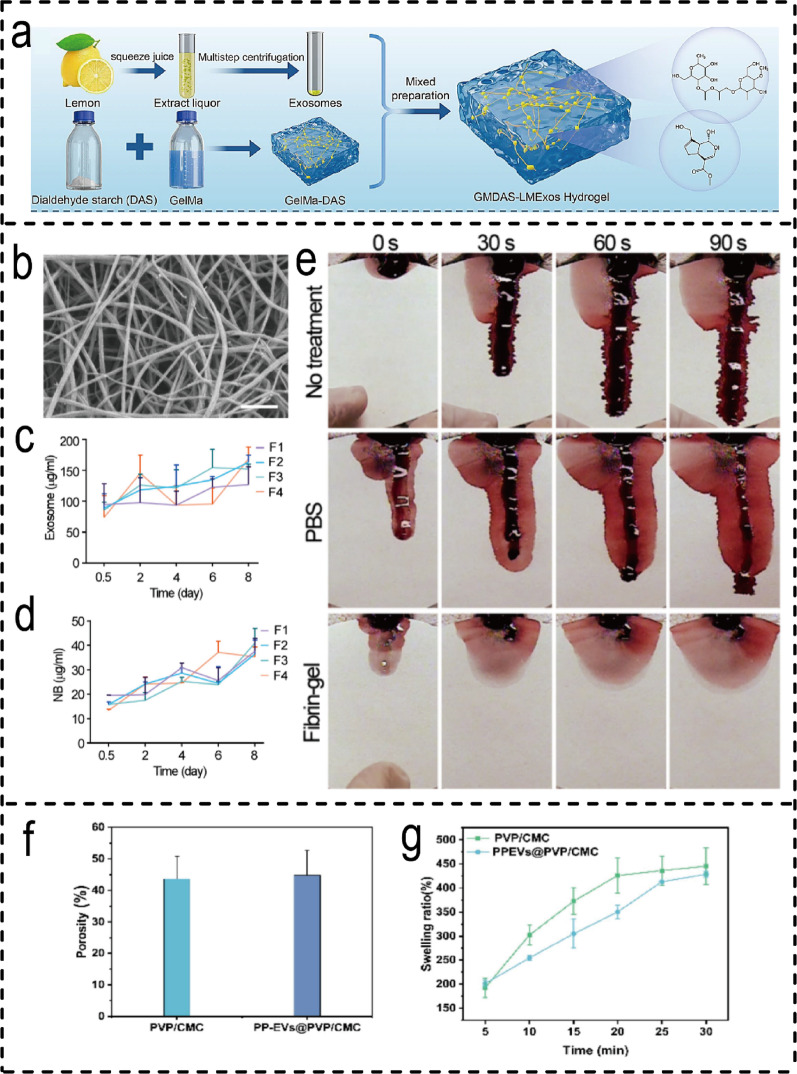
Types of hydrogels for Plant-Derived EVH systems. (**a**) Preparation of the lemon exosome hydrogel [203]. (Copyright 2025, Springer Nature.) (**b**) SEM images of Fibrin-gel. (**c**) Release of Cy5.5-labeled ELNs in the Fibrin-gels. (**d**) Release of NB in the Fibrin-gels. (**e**) Photograph of hemostasis process [204]. (Copyright 2024, Elsevier.) (**f**) Porosity of PVP/CMC and PPEVs@PVP/CMC hydrogels (*n* = 3). (**g**) Swelling ratio curves of PVP/CMC and PPEVs@PVP/CMC hydrogels (*n* = 3) [205]. (Copyright 2025, Elsevier.)

Plant-derived EVs are garnering increasing attention in the field of wound healing due to their unique biological activity. An increasing number of Plant-EV-combined hydrogel therapeutic systems have been proposed by researchers. For example, natural hydrogels demonstrate outstanding performance: for instance, gelatin methacrylate-bisacetaldehyde starch (GelMA-DAS) hydrogels not only provide a moist yet breathable wound interface but also enable sustained release of lemon-derived exosomes, maintaining therapeutic efficacy in diabetic wound models [203] (Fig. 10a**)**. Fibrin-based hydrogels demonstrate rapid in situ gelation and exceptional haemostatic performance, achieving haemostasis within 31 s in mouse models [204] (Fig. 10b), whilst enabling sustained co-release of rose-derived exosome-like nanoparticles (ELNs) and antibiotics for up to eight days (Fig. 10c, d). Synthetic and composite hydrogels offer further functional customisation potential. For instance, the polyvinylpyrrolidone/carboxymethyl chitosan (PVP/CMC) hydrogel W exhibits high porosity (43.6 ± 7.2%) and swelling capacity (45.4 ± 38.3%), facilitating rapid exudate absorption and controlled vesicle release [205] (Fig. 10e, f, g), though its highly porous structure may compromise mechanical strength. These cases reveal a critical design trade-off: natural hydrogels (e.g., fibrin) offer advantages in biocompatibility and rapid haemostasis, whilst synthetic or hybrid systems (e.g., PVP/CMC, GelMA-DAS) excel in tunable drug release and structural integrity. However, further optimisation remains necessary to balance elasticity and long-term functional durability within dynamic wound environments [203, 205].

#### Molecular mechanisms of plant-EVH in promoting wound healing

**Fig. 11:**
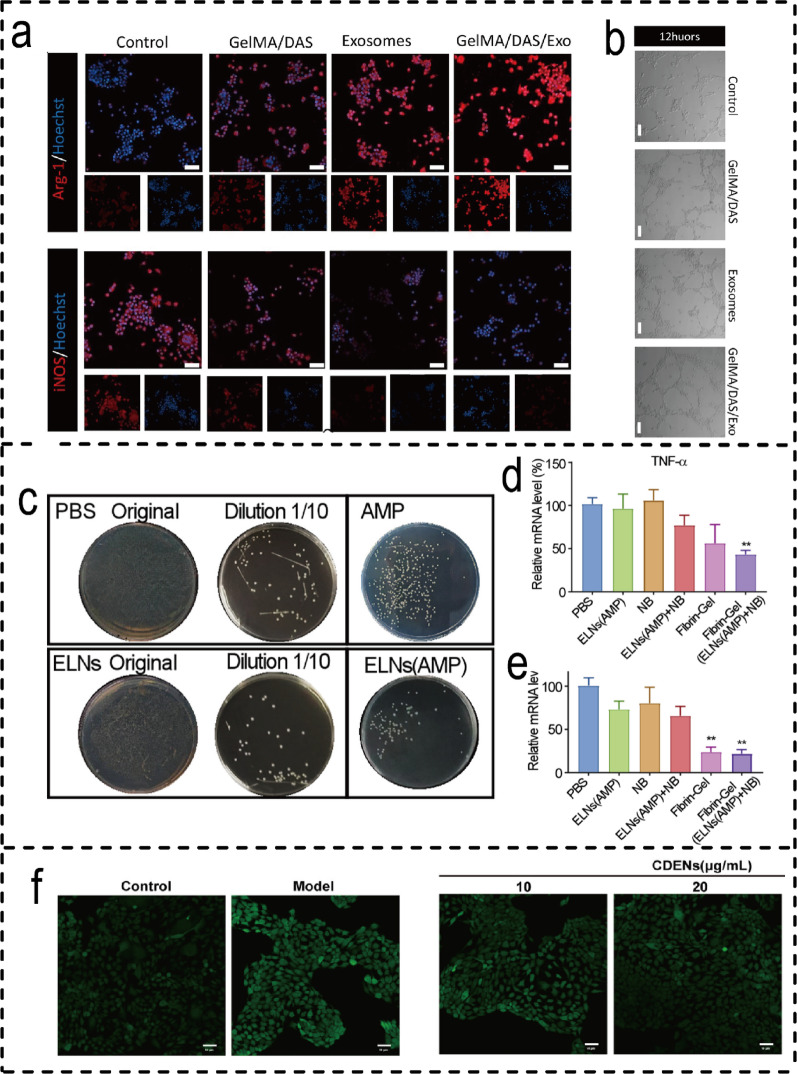
Mechanism of action of Plant-EVH Systems. (**a**) IF images showing the number of Arg1 (red)-positive and iNOS (red)-positive RAW264.7 cells cultured on each hydrogel. The scale bar represents 50 μm. (**b**) Vessel formation analysis of HUVECs cultured on different materials for 12 h [203]. (Copyright 2025, Springer Nature.) (**c**) Photos of intracellular MRSA with different treatments. (**d-e**) The mRNA levels of TNF-α, IL1β [204]. (Copyright 2024, Elsevier.) (**f**) The fluorescence images of intracellular ROS levels visualized by CLSM (scale bar, 50 μm) [206]. (Copyright 2025, John Wiley and Sons.)

**Fig. 12:**
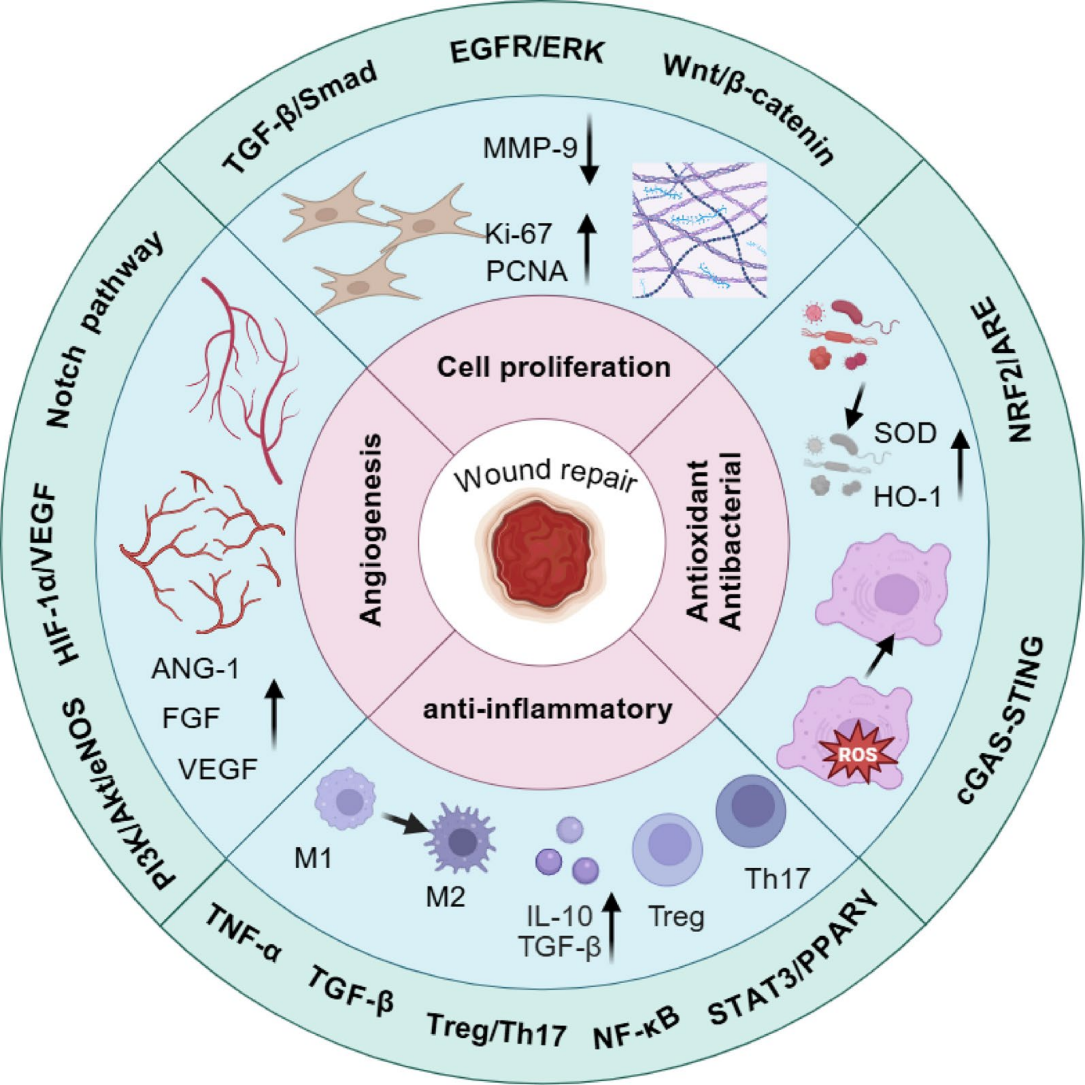
Mechanisms by which EVH promotes wound healing(Figure created using BioRender)

Plant-derived EVH promote wound healing through synergistic mechanisms including immunomodulation, antioxidant effects, and regeneration promotion. Among these, lemon-derived EVH directly induce M2 macrophage polarisation (Fig. 11a) and enhance vascular endothelial cell migration (Fig. 11b), accelerating diabetic wound repair [203]. rose-derived EVH, when co-loaded with antimicrobial peptides, exhibit a 2.5-fold increase in intracellular antibacterial activity (Fig. 12c). Within fibrin hydrogels, they synergistically reduce pro-inflammatory factor levels (TNF-α, IL-1β) alongside antibiotics (Fig. 11d, e), thereby promoting granulation tissue formation [204]. Regarding antioxidant effects, coriander-derived EVH scavenge ROS by activating the Nrf2 pathway (Fig. 11f), coordinating multiple stages from inflammation resolution to tissue reconstruction [206]. Conversely, the anti-inflammatory and ROS-scavenging efficacy of Paris polyphylla EVs relies entirely on encapsulation within PVP/CMC hydrogels to achieve local retention and controlled release [205]. These cases demonstrate that integrating the intrinsic activity of plant-EVs with material delivery systems can effectively promote wound healing, though release kinetics and stability require further optimisation.

### Other-Derived EVH Systems for Wound Healing

Other sources of EVs have also been incorporated into hydrogels by researchers for wound healing applications. By encapsulating these EVs within hydrogels—such as GelMA, chitosan-based thermosensitive gels, sericin hybrids, DNA micro-flower networks, and 3D bioprinted scaffolds—these systems enable sustained EV release, improved targeting, and enhanced functional stability at the wound site. (Table 5).

**Table 5:** Comparing the biological functions of EVH systems formed by EVs from various sources

Sources of EVs	Hydrogel Type	Key Functions	Mechanism / Pathway	Ref.
Platelets	• Methacrylated acellular dermal matrix (ADMMA-GEL) • DNA microflower hydrogel	• Anti-inflammatory • Pro-angiogenic • Bacteria-responsive	• M2 macrophage polarization and neovascularization • Bacterial-triggered degradation and controlled release	[129, 207]
Hypoxic USCs	Decellularized ECM hydrogel (SISMA)	Angiogenesis, wound closure	miR-486-5p targeting SERPINE1, HIF-1α activation	[173]
Fibroblasts	Gelatin methacryloyl (GelMA)	Epithelialization, vascularization, anti-fibrosis	miRNA-mediated cell migration and differentiation	[113]
Hemangioma stem cells	Chitosan/hyaluronic oligosaccharide thermosensitive gel (oHA/CSgel)	Microcirculation reconstruction, angiogenesis	miRNA-targeted angiogenic processes	[208]
NIH/3T3 cells (si-TNF-α-EVs)	3D-bioprinted core–sheath scaffold	Anti-inflammatory, M2 polarization	siRNA-mediated TNF-α suppression	[209]
Platelet-rich plasma	Pluronic F127 thermosensitive hydrogel	Anti-ferroptosis, anti-inflammatory	FosB-mediated GPX4/SLC7A11/ACSL4 regulation	[210]
Epidermal stem cells (VH298-EVs)	GelMA hydrogel	Angiogenesis, wound healing	HIF-1α/VEGFA signaling activation	[211]
Dental pulp stem cells	Silk nanofibril/Halloysite nanotube hybrid gel	Hemostasis, antimicrobial, pro-angiogenic	Angiogenesis and collagen promotion	[212]
Foreskin MSCs	Injectable PVP-silicotungstate hydrogel (PSiW)	Macrophage polarization, angiogenesis	Microenvironment remodeling	[158]
Neutrophils	Extracellular matrix (ECM) hydrogel	Antibacterial, pro-angiogenic, immunomodulatory	VEGF delivery, immune regulation	[213]
Royal Jelly	Photocrosslinked Sericin Methacrylate (SerMA) Hydrogel	• Anti-inflammatory • Antioxidant • Promotes cell proliferation & migration (L929 cells) • Accelerates wound healing (96.8% healing rate) • Promotes angiogenesis	• Involved in recombinational repair • Regulates epidermis development • Activates Wnt signaling pathway	[214]

### Comparison, selection and design principles for EVH systems from different sources

#### Composition, performance and efficacy comparison of the EVH system

To provide an intuitive comparison of design strategies and therapeutic efficacy across different systems, this section synthesises literature across five dimensions: EVs source, hydrogel matrix, EV-derived haemoglobin characteristics, therapeutic mechanisms, and in vivo efficacy. Findings indicate that system design exhibits an evolutionary trend towards ‘function-oriented’ and ‘intelligent synergistic’ approaches. Specifically, EV source determines functional orientation: MSC/ADSC-derived EVs serve as the most widely applied ‘multipotent tools’. Immune cell-derived EVs (e.g., M2-EVs) and platelet-derived EVs provide targeted immunomodulation. Plant-derived EVs offer advantages in cost and antioxidant properties. Engineered EVs represent a frontier development direction. Hydrogel matrices influence delivery strategies, with natural polymers (e.g., GelMA, chitosan, alginate) being mainstream choices due to their excellent biocompatibility [107, 115]. Smart responsive hydrogels (e.g., ROS-, pH-, or enzyme-responsive) enable on-demand EV release, better adapting to the dynamic wound microenvironment [65, 150]. injectable thermosensitive hydrogels (PF-127) significantly enhance clinical operational convenience [142, 215]. EVH characteristics correlate closely with therapeutic efficacy. Systems integrating sustained/controlled release with antimicrobial, antioxidant, and immunomodulatory functions [183, 216] demonstrate superior healing outcomes when addressing the complex pathologies of chronic wounds, embodying the advanced concept of integrated synergistic therapy**(**Fig.12 **)**.

#### Assessment of the strength of evidence for research into the mechanism of action of EVH therapy

We conducted a tiered assessment of evidence for key molecular pathways. Evidence was categorised into three levels based on whether functional gain/loss experiments validated the causal relationship between specific molecules in EVs and phenotypes: Level I evidence (strong causal evidence) required direct validation through gene knockdown/overexpression, specific inhibitor blockade, or key molecule rescue experiments, such as studies established a complete mechanistic chain by constructing miR-17-5p-overexpressing sEVs, performing functional rescue after miR-494-3p knockout [157], or employing pathway inhibitors [134]. Level II evidence (moderate associative evidence) is prevalent in most studies, typically involving the identification of differentially expressed molecules via sequencing followed by in vitro functional validation, or demonstrating pathway activation through WB/qPCR without direct intervention verification of the molecule [115, 144, 215]. Level III evidence (descriptive/hypothesis-based mechanisms) primarily relies on literature-based speculation or preliminary omics analyses without validation. Overall, most research in this field remains at Level II evidence, providing a correlational foundation for mechanistic hypotheses. Future efforts should strengthen functional rescue experiments, particularly by applying engineered EVs in animal models, thereby advancing mechanistic claims from ‘correlation’ to ‘causation’.

#### Performance Comparison and Advantages/Disadvantages of Different EVH Systems

**Table 6:** Representative EVH system configuration and performance comparison

EV Source	Hydrogel	Key Features of EVH	Mechanism of action	Ref.
**MSC**	GelMA, CS, Alg, PF-127	Promotes angiogenesis and collagen deposition, and regulates macrophage M2 polarisation	PI3K/Akt, VEGF signalling pathway, miR-17-5p/PTEN/p21 (engineered)	[107, 109, 115, 119, 215]
**ADSC**	GelMA, Dynamic Network Hydrogels	Engineering strategies (such as miRNA overexpression) may enhance therapeutic efficacy	Akt, PI3K/AKT, regulates ageing and mitochondrial function	[163, 217–219]
**M2 macrophages**	Multifunctional hydrogel, HA/PVA/2-FPBA/PEI (Microneedling)	Directly delivers immunomodulatory signals to reshape the anti-inflammatory microenvironment	Provide anti-inflammatory cytokines to induce M2 polarisation	[73, 145, 179]
**HUVECs**	GelMA, ADM/GelMAComposite hydrogel	Targeting angiogenesis, hypoxic pre-treatment can enhance its efficacy	PTEN/PI3K/AKT pathway promotes endothelial cell proliferation and migration	[178, 214, 220]
**Platelets**	Composite hydrogel (GelMA/SFMA)	Rich in growth factors, it powerfully promotes haemostasis, angiogenesis and cell proliferation	Transmit growth factors (PDGF, TGF-β) and regulate inflammation	[162, 216]
**Plants**	Natural/Composite Hydrogels (CS, Alg)	Widely sourced and low-cost, it possesses antioxidant, anti-inflammatory and tissue-repairing properties	ROS scavenging (Nrf2/HO-1), immunomodulation	[113, 221–223]
**Engineering/Other sources**	Customisable smart hydrogels (e.g. ROS/pH responsive)	Achieving functional enhancement and targeted delivery through payload loading (drugs, miRNAs), surface modification, or culture optimisation	Mechanisms are highly customisable, such as inhibiting ferroptosis/copper-induced cell death and activating specific miRNA pathways	[137, 165, 183]

We compared the various systems across dimensions including release characteristics, wound-healing efficacy, and clinical translation feasibility **(**Table 6**)**. Our analysis indicates that the ‘intelligent response’ systems represent the future direction in terms of release precision and microenvironment adaptability [65, 150, 217]. The ‘multifunctional integrated’ system simultaneously addresses multiple barriers—infection, oxidation, inflammation, and regeneration—through a single dressing, offering an effective strategy for managing the complexity of diabetic wounds [183, 218]. While the ‘engineered EV’ strategy significantly enhances core therapeutic efficacy, it faces challenges in standardisation and regulatory compliance [119, 217]. Conversely, simple, user-friendly systems demonstrate greater feasibility during early-stage translation [215]**(**Table 7**)**.

**Table 7:** Comparative analysis of key performance characteristics in the EVH system

Comparison dimension	Advantages	Limitations/Challenges	Ref.
**Release dynamics**	**Intelligent Release Mechanism**: Responding to the wound microenvironment (reactive oxygen species, pH, glucose, enzymes) to achieve on-demand, precise release, enhancing EV utilisation and adapting to the requirements of different healing stages	**Passive/Sustained Release**: The release curve (such as that of a burst-release effect) may not perfectly match dynamically evolving pathological processes, potentially leading to initial wastage or subsequent supply insufficiency	[65, 109, 150, 167]
**EV Load and Protection**	**High-load/Stability Strategy**: Enhance loading efficiency and preserve EV biological activity through chemical conjugation, affinity interactions (e.g., polyphenol-protein), or encapsulation within microspheres/networks	**Simple physical mixing**: Limited loading efficiency, with the active ingredient prone to inactivation or premature leakage during gel formation due to physicochemical stresses	[107, 215–217]
**Angiogenic efficacy**	**Engineered/targeted EVs**: Through genetic engineering to overexpress pro-angiogenic miRNAs (e.g., miR-17-5p) or surface-modified targeting ligands (e.g., sLeX), significantly enhancing regulatory efficacy and specificity towards endothelial cells	**Ordinary natural EV**: Its vasoactive potential may be limited, particularly in diabetic wound microenvironments characterised by severe ischaemia or high inflammation, where it struggles to achieve optimal efficacy.	[119, 215, 219]
**Immune Modulation and Anti-Inflammatory Effects**	**Specific cell-derived EVs/synergistic regulation**: Directly utilise EVs derived from M2 macrophages or Treg cells to deliver explicit anti-inflammatory signals; or leverage the antioxidant properties of hydrogels to synergistically promote M2 polarisation.	**A single anti-inflammatory component**: may prove insufficient to reverse the persistent chronic inflammatory state in diabetic wounds, which is driven by multiple factors.	[126, 144, 150]
**Resistance to infection**	**Integrated antimicrobial components**: Incorporating inherent antimicrobial materials (such as quaternised chitosan, ε-polylysine) or loaded antimicrobial agents (AgNPs, antibiotics) within hydrogels to proactively prevent or treat wound infections	**Systems without antimicrobial function**: Limited application in wounds with existing or high-risk infections, requiring reliance on systemic or additional topical antimicrobial therapy	[153, 216, 220]
**Clinical translation convenience**	**Injectable/self-healing/user-friendly**: Simple to administer (e.g. in situ injection, spray application), conforms to irregular wound surfaces; relatively straightforward preparation process with good reproducibility, readily scalable	**Complex engineering/requires external triggering**: Production processes are intricate, costly, and challenging to quality control; certain systems necessitate external triggering conditions (such as UV light), thereby limiting their application scenarios and convenience	[119, 183, 215, 221]

#### Key parameter analysis and outlook for clinical translation

Based on systematic comparisons, we have identified key parameters and future directions for advancing the clinical application of EVH systems: Firstly, establishing a standardised quality control system is paramount. Current research exhibits significant variations in EV isolation, characterisation, and loading doses, necessitating unified standardised protocols for production, characterisation, and release performance assessment [109, 115]. Secondly, enhanced safety and long-term biocompatibility assessments are required, particularly concerning the long-term toxicology, immunogenicity, and degradation product effects of complex systems (e.g., synthetic materials and engineered EV). Furthermore, scalable production and cost-effectiveness are paramount: simplified systems based on natural polymers offer advantages in cost and scalability, whilst complex intelligent systems necessitate solutions for process scaling and cost control [119]. Finally, predictability and personalisation of therapeutic outcomes warrant attention. To address the heterogeneity of diabetic wounds, future systems may evolve towards ‘modular’ or ‘multi-stage response’ models, enabling customised combinations of functional modules—such as antimicrobial, anti-inflammatory, and pro-angiogenic components—tailored to the specific wound condition.

At present, multifunctional integration and intelligent response design have become mainstream strategies for addressing the complex microenvironments of chronic wounds. To achieve genuine clinical translation, future efforts must focus on: (1) Strengthening Level I evidence by solidifying theoretical foundations through rigorous causal experiments. (2) Conducting head-to-head comparative studies to clarify the relative advantages and disadvantages of various design strategies across different animal models. (3) Overcoming translational medicine bottlenecks by achieving breakthroughs in standardised, large-scale production, long-term safety evaluation, and personalised treatment protocol design. The integrated analytical framework developed in this study aims to provide a clear ‘roadmap’ for peers to select optimised pathways and position research value.

## Translational medicine perspective: challenges and prospective analysis from preclinical research to clinical application

**Fig. 13:**
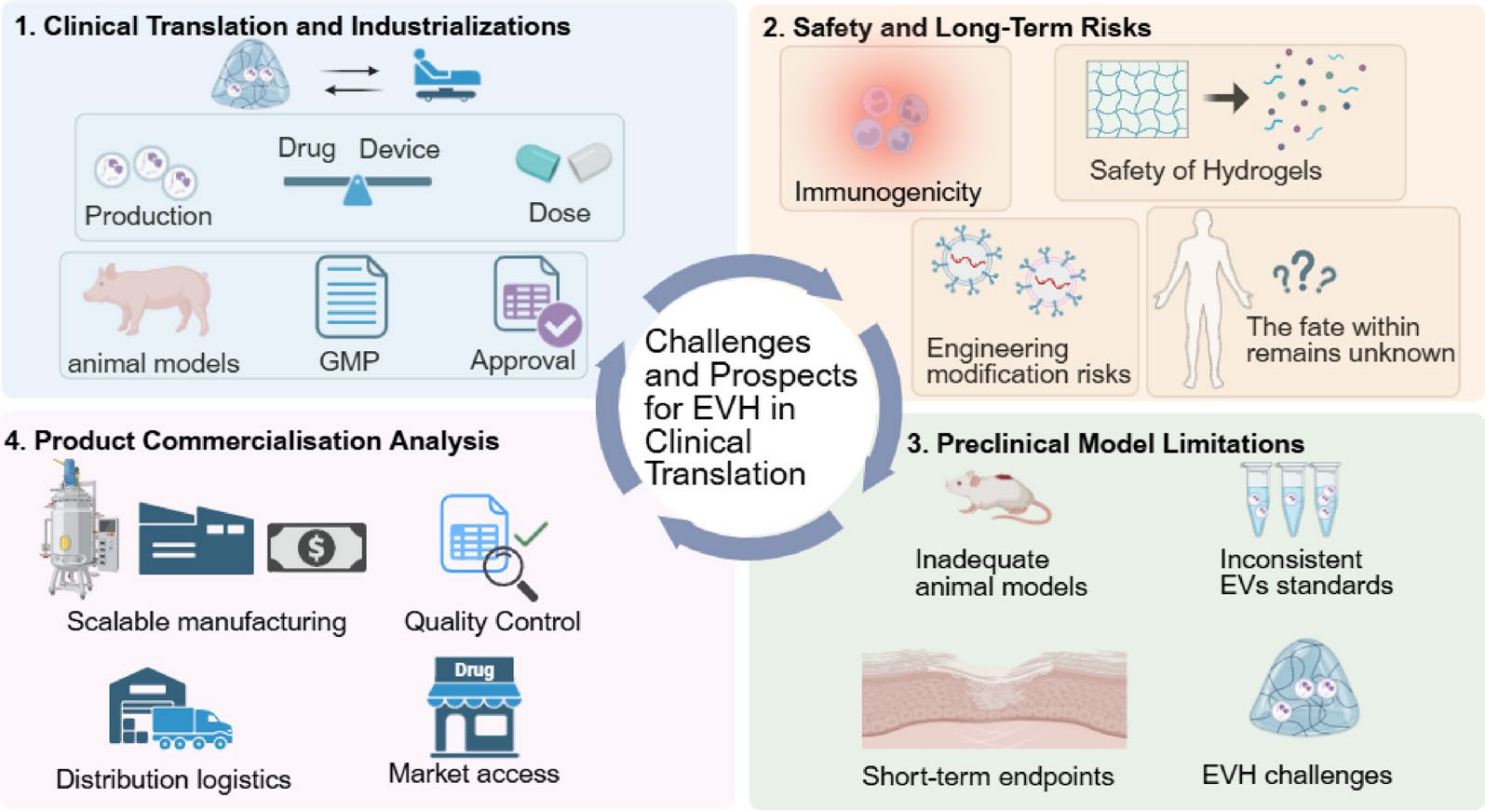
Schematic Diagram of Clinical Translation Challenges(Figure created using BioRender)

Despite demonstrating considerable potential for promoting wound healing in preclinical studies, the EVH system faces a series of complex scientific, regulatory, and industrialisation challenges in advancing towards genuine clinical application. This chapter aims to systematically analyse the translational medicine barriers within this field, critically evaluate the limitations of existing research, and conduct an in-depth examination of regulatory pathways, safety, production feasibility, and commercial prospects. This endeavour seeks to provide a clear roadmap for future research. **(**Fig. 13)

### Clinical translation of the EVH system: status, challenges and pathways

Despite the significant potential of EVH systems demonstrated in preclinical wound healing research, their journey to clinical application faces substantial translational barriers [128, 222]. At present, the preponderance of extant evidence remains constrained to small animal models, with a marked paucity of registered clinical trials for specific EVH products, signifying a critical transition phase from proof-of-concept to clinical implementation (Table 8).

The three core challenges are as follows. Firstly, significant challenges are evident in the technical and manufacturing domains. The large-scale production of EVs is hindered by low yield, batch-to-batch heterogeneity, and a lack of standardized potency assays and functional characterization criteria [27, 124, 128]. Secondly, the regulatory complexity inherent to the combination of a biological agent and medical device in EVH necessitates adherence to dual regulatory pathways, thereby complicating the development and approval processes [123, 223]. A translational gap has been identified, which is attributable to insufficient validation in clinically relevant large animal models, a paucity of long-term safety data, and undefined optimal dosing regimens [138, 224, 225].

In order to facilitate clinical translation, a structured development pathway is imperative. The following elements are to be considered: Firstly, the utilisation of large animal models is imperative for conducting systematic safety and efficacy assessments during the preclinical phase [27, 224]. Secondly, the establishment of GMP-compliant, scalable manufacturing processes and comprehensive quality control standards for the final combination product is essential [117, 226]. Thirdly, the design of clinical trials should prioritise safety and dose exploration, followed by confirmatory studies employing clinically relevant endpoints such as complete wound closure rates [227, 228]. It is imperative that proactive engagement with regulatory authorities is initiated at the outset in order to successfully navigate the complex approval pathway [138].

**Table 8:** Clinical trials of EVH for wound healing

Trial ID	EVs source	Delivery Method	Phase	Result
NCT02565264	Autologous plasma	Direct application	Ⅰ	No results posted
NCT04134676	WJ-MSC Conditioned Medium	Topical gel + transparent dressing	Ⅰ	No results posted
NCT06825884	Platelets	Perilesional injection	Ⅰ	No results posted (Ongoing)
NCT06812637	WJ-MSC	CMC-based gel	Ⅰ	No results posted (Study completed 2024-09-02)
NCT05078385	Bone marrow derived mesenchymal stem cells	No posted	Ⅰ	No results posted(Ongoing)
NCT06319287	Platelets	EVs encapsulated in TISSEEL fibrin sealant	Ⅱ	No results posted(Staudy completed 2025-11-01)
ChiCTR2500103555	Human umbilical cord mesenchymal stem cells	Subcutaneous injection	Ⅰ/Ⅱ	No results posted(Date of Registration:2025-05-30)
ACTRN12620000944932	platelets	Subcutaneous injection	Ⅰ	Good biological safety

### Biosafety considerations and long-term risk assessment

A comprehensive and rigorous biosafety assessment is a prerequisite for the clinical translation of EVH systems, an area currently overshadowed by a predominant focus on efficacy in research [115, 132, 144, 171]. While preliminary studies frequently report an absence of significant acute toxicity or major organ damage in animal models [112, 115, 121], these findings are insufficient to guarantee comprehensive safety.

Safety risks must be examined at multiple levels: firstly, the EVs themselves, It is imperative that potential risks be evaluated at multiple levels. The potential risks associated with EVs include the possibility of immune responses triggered by allogeneic or xenogeneic sources, engineering modifications, or repeated high-dose administration [27, 123, 132, 229, 230]. The risks associated with hydrogel carriers are contingent on material degradability; slow-degrading synthetic or composite materials have the potential to induce chronic inflammation, while incorporated nanomaterials (e.g., silver nanoparticles) can introduce concerns regarding toxicity and long-term accumulation [153, 155, 165, 196, 231, 232].

The risks associated with complex systems are amplified in engineered designs, such as genetically modified EVs, which pose genotoxic risks [119, 217, 233], or hybrid systems, which exhibit unpredictable in vivo behaviour [121, 213, 234]. Furthermore, ‘smart’ release systems reliant on microenvironmental responses may become uncontrolled within complex, dynamic wound environments, leading to unintended release [16, 149]. Collectively, these factors heighten the uncertainty and risk profile of EVs-based therapeutic systems.

The most critical knowledge gap pertains to long-term systemic safety. The biodistribution, off-target effects, and potential impact of EVs on distant organs or pre-existing conditions (e.g., stimulating dormant tumour cells) remain poorly understood [27, 114, 123, 128]. The prevailing evaluation models are inadequate for the purposes of assessing chronic toxicity, immunogenicity, or carcinogenic potential [235, 236]. A paradigm shift towards a “safety-first” approach is imperative, necessitating long-term toxicology studies, advanced in vivo tracking, and the establishment of standardized safety and quality control thresholds [27, 134–136, 166, 221, 237].

### Limitations of preclinical models and standardization hurdles

The preclinical basis for EVH therapy is considerably constrained by the absence of standardisation and the clinical inadequacy of animal models. However, the extensive utilisation of streptozotocin-induced type 1 diabetic rodent models has proven to be inadequate in accurately replicating the multifaceted pathophysiology of human type 2 diabetic wounds, which frequently encompass components such as ischemia and neuropathy [134, 165, 238]. The heterogeneity of wound parameters, in conjunction with a predominant focus on short-term histological endpoints, further diminishes the clinical predictive value of these studies [115, 152, 238–241].

A significant translational barrier pertains to the absence of unified pharmacological standards for EVs. Inconsistent reporting of dosage metrics (e.g. mass, particle count, volume) and a paucity of systematic dose-response studies are pervasive [115, 119, 242, 243]. It is important to note that there is no standard bioassay to quantify EV “potency,” which prevents meaningful comparison between EVs from different sources or preparations [111, 161, 244, 245]. EVs) are frequently regarded as a homogeneous black box, with inadequate analysis of functionally critical subpopulations and specific cargo, resulting in an underdefinition of their structure-function relationship [119, 134, 138, 246].

The mechanistic understanding of the EV-hydrogel-wound microenvironment interplay is underdeveloped. While the sustained release of hydrogels has been characterised in vitro [115, 116, 177], the release kinetics under pathologically relevant wound conditions and the real-time in vivo bioavailability of EVs remain a subject of investigation [138, 221, 238]. The impact of encapsulation on EVs integrity and the deeper synergistic mechanisms by which hydrogels modulate the local microenvironment to enhance EVs function remain largely unexplored [65, 144, 216, 238].

### Industrialization and Commercialization Pathways

The process of translating EVH from a laboratory prototype to a marketable product gives rise to systemic industrial challenges. The primary bottleneck is the establishment of scalable, cost-effective, and GMP-compliant manufacturing processes for both biological (EVs) and material (hydrogel) components, and their subsequent combination [115, 151, 175]. The complexity of the process has been demonstrated to directly impact cost, thus rendering treatment affordability a pivotal factor in determining commercial viability [134, 247, 248].

The establishment of a comprehensive quality control (QC) system is imperative for the attainment of regulatory approval. In the context of EVH, QC must encompass the entire product life cycle, from raw materials to the final product, necessitating meticulous characterisation of EVs attributes, hydrogel properties, and composite performance [116, 221]. Furthermore, the overcoming of logistical hurdles related to EVs stability is critical for distribution. Such hurdles may be overcome through the development of stable formulations (e.g., lyophilized powders) and a reliable cold chain [221, 249, 250].

The concept of successful commercialisation extends beyond the realm of manufacturing. In order to achieve this, it is necessary to navigate the complex landscape of intellectual property and formulate clear market access strategies. These strategies must include targeting high-need indications, designing viable pricing and reimbursement models, and utilising expedited regulatory pathways [134, 247, 248]. In view of the multifaceted challenges, strategic collaborations across industry, academia, and healthcare are imperative in order to share risks and accelerate the translation of EVH from a promising platform to an accessible clinical therapy.

## Conclusion

The integration of hydrogels and EVs represents a promising regenerative strategy, effectively overcoming key limitations of EVs monotherapy—such as rapid clearance and enzymatic degradation—by providing localized, sustained, and microenvironment-responsive release. This synergistic system has demonstrated significant potential in enhancing angiogenesis, immunomodulation, and tissue repair in chronic wound models. Moving forward, efforts should focus on standardizing EVs production, improving hydrogels tunability for spatiotemporally controlled release, and elucidating underlying molecular mechanisms to facilitate clinical translation.

## Data Availability

No datasets were generated or analyzed during the current study.

## Abbreviations

EVs: Extracellular vesicles
EVH: Extracellular vesicles-Hydrogels
PEG: Polyethylene glycol
PVA: Polyvinyl alcohol
ROS: Reactive oxygen species
ADSCs: Adipose-derived stem cells
HUCMSCs: Human umbilical cord mesenchymal stem cells
BMSCs: Bone marrow-derived mesenchymal stem cells
MSC: Mesenchymal stem cells
SHED: Human exfoliated deciduous Teeth Stem cell
MMP: Matrix Metalloproteinases
VEGF: Vascular endothelial growth factor
RGD: Arginine-Glycine-Aspartic acid
GelMA: Gelatin Methacryloyl
PRP: Platelet-rich plasma
DPSCs: Dental pulp stem cells
USCs: Urine-derived stem cells
ISEV: International Society for Extracellular Vesicles
MISEV: Minimal information for studies of extracellular vesicles
GMP: Good Manufacturing Practice
